# *Etv2-miR-130a-Jarid2* cascade regulates vascular patterning during embryogenesis

**DOI:** 10.1371/journal.pone.0189010

**Published:** 2017-12-12

**Authors:** Bhairab N. Singh, Naoyuki Tahara, Yasuhiko Kawakami, Satyabrata Das, Naoko Koyano-Nakagawa, Wuming Gong, Mary G. Garry, Daniel J. Garry

**Affiliations:** 1 Lillehei Heart Institute Regenerative Medicine and Sciences Program, University of Minnesota, Minneapolis, MN, United States of America; 2 Department of Genetics, Cell Biology and Development, University of Minnesota, Minneapolis, MN, United States of America; 3 Paul and Sheila Wellstone Muscular Dystrophy Center, University of Minnesota, Minneapolis, MN, United States of America; Medical College of Wisconsin, UNITED STATES

## Abstract

Remodeling of the primitive vasculature is necessary for the formation of a complex branched vascular architecture. However, the factors that modulate these processes are incompletely defined. Previously, we defined the role of *microRNAs* (*miRNAs*) in endothelial specification. In the present study, we further examined the *Etv2-Cre* mediated ablation of *Dicer*^*L/L*^ and characterized the perturbed vascular patterning in the embryo proper and yolk-sac. We mechanistically defined an important role for *miR-130a*, an Etv2 downstream target, in the mediation of vascular patterning and angiogenesis *in vitro* and *in vivo*. Inducible overexpression of *miR-130a* resulted in robust induction of vascular sprouts and angiogenesis with increased uptake of acetylated-LDL. Mechanistically, *miR-130a* directly regulated *Jarid2* expression by binding to its *3’-UTR* region. Over-expression of Jarid2 in HUVEC cells led to defective tube formation indicating its inhibitory role in angiogenesis. The knockout of *miR-130a* showed increased levels of *Jarid2* in the ES/EB system. In addition, the levels of *Jarid2* transcripts were increased in the *Etv2-null* embryos at E8.5. In the *in vivo* settings, injection of *miR-130a* specific morpholinos in zebrafish embryos resulted in perturbed vascular patterning with reduced levels of endothelial transcripts in the *miR-130a* morphants. Further, co-injection of *miR-130a* mimics in the *miR-130a* morphants rescued the vascular defects during embryogenesis. qPCR and *in situ hybridization* techniques demonstrated increased expression of *jarid2a* in the *miR-130a* morphants *in vivo*. These findings demonstrate a critical role for *Etv2-miR-130a-Jarid2* in vascular patterning both *in vitro* and *in vivo*.

## Introduction

Endothelial precursors (angioblasts) emerging from the nascent mesoderm proliferate, migrate and coalesce to form the primitive vascular plexus [1]. Remodeling of the primitive vasculature is necessary for the formation of a complex vasculature by the process of angiogenesis [1, 2]. Formation of blood vessels is a complex multistep process that requires precise control and coordination of endothelial cell behavior and other environmental cues [1, 3, 4]. Transcriptional networks and signaling pathways that regulate these processes are not completely known [3, 5, 6].

Given the complexity of the vascular system, a variety of factors and pathways are known to be involved in the modulation of the angiogenic response [1, 3, 7]. *Etv2* (Ets-family transcription factor) is expressed in the earliest endothelial progenitors and shown to be essential for both endothelial and hematopoietic lineages during development [8–10]. Studies have shown that Etv2 plays a critical role in mesodermal lineage specification by modulating the transition of Flk1^+^/Pdgfra^+^ primitive mesodermal lineages [11]. In the absence of Etv2, the formation of Flk1^+^/Pdgfra^−^ lateral plate mesodermal cell population is perturbed during embryogenesis [11, 12]. The global knockout of Etv2 results in embryonic lethality by E9.5 due to the complete absence of the hematopoietic and endothelial lineages [8, 9, 11]. Previous studies have established that *Etv2* gene expression is modulated by Wnt, BMP and Notch signaling factors to regulate hematopoiesis [13]. Multiple studies have demonstrated that regulation of the hematopoietic and endothelial lineage by Etv2 is mediated through its interaction with Gata2, Scl, Lmo2, Tie2, Foxc2 and Vegf signaling [5, 9, 13–16]. We and others have shown that the hierarchical relationship between Etv2, Vegf, p38 MAP kinase and CREB signaling in the modulation of the hematopoietic and endothelial lineages [15–17]. Together these reports establish that Etv2 is a master regulator of hematopoietic and endothelial lineage development [18–20].

Several growth factors and signaling cascades including VEGF, FGF, SHH and MAPkinase have been shown to promote angiogenesis by promoting endothelial cellular (EC) proliferation, cell-cell interaction and migration during development and patho-physiological conditions [21–24]. Other factors including PDGFR-β are indispensable for the synergistic effect of VEGF-A and FGF-2 on neoangiogenesis in adults [21]. Deciphering additional factors that regulate vascular development and angiogenesis would be of intense interest for the field. While the transcriptional regulation of endothelial development is documented, the role of other regulatory mechanisms, including miRNAs, during vascular development is unknown.

*MicroRNAs* (*miRNAs*) are a class of small non-coding RNA that suppress gene expression by targeting mRNAs at the *3’-UTR* for cleavage and/or degradation [25]. More than 1000 *miRNAs* have been identified in mammals that are involved in critical steps during development and pathological conditions [26]. Large *pre-miRNAs* are processed in multiple steps to give rise to a mature *miRNA*. Maturation of *pre-miRNAs* into a mature form (~20–22 nucleotides) is mediated by *Dicer*, a *miRNA* processing enzyme [27]. Global deletion as well as hypomorphic mutants (deleting exon 1 and 2) of *Dicer* results in embryonic lethality [27, 28]. Previously, we combinatorially mated *Etv2-Cre* and *Dicer*^*L/L*^ mice to generate conditional *Dicer* knockouts [29]. These studies identified *miR-130a* as an essential factor in the specification of the endothelial lineage [29]. Furthermore, *miR-130* was identified as the first factor to specifically promote hematopoietic and endothelial progenitor divergence to the endothelial lineage [29]. In the present study, we determined that the loss of *Dicer* in the endothelial precursors resulted in defective vascular remodeling and patterning. We further defined an additional role for *miR-130a* as an important regulator of angiogenesis and vascular patterning both *in vitro* and *in vivo* via its regulation of *Jarid2*.

## Materials and methods

### Generation of *Dicer* conditional null mice

All studies using animals were approved by the Institutional Animal Care and Use Committee of the University of Minnesota. We characterized the lethality of the conditional knockout embryos by examining the progeny of *Etv2*^*Cre/+*^*;Dicer*^*L/+*^ and *Dicer*^*L/L*^ mice. Genotyping was performed using specific PCR primers and standard procedures.

### Morphological analysis and whole-mount staining

For morphological analysis, embryos from time-mated females were harvested at distinct stages and imaged using the Zeiss Axio Observer Z1 inverted microscope. Whole-mount staining for the endothelial architecture was performed as described [11, 30]. Briefly, time-mated embryos were fixed in 4% paraformaldehyde (PFA) at 4°C for 6 h, washed in PBT (PBS, 0.1% Tween 20) followed by dehydration through a graded series of methanol (25%, 50%, 80%, and 100%). Dehydrated embryos were rehydrated in decreasing methanol concentration and the endogenous peroxidase activity was quenched with 3% H_2_O_2_. Embryos were washed in Pblec [PBS, 1% Tween 20, 1 mM CaCl_2_, 1 mM MgCl_2_, and 0.1 mM MnCl_2_ (pH 6.8)], and incubated with rat anti-endomucin antibody (1:30 dilution; in blocking buffer), followed by incubation with anti-rat Cy3-conjugated secondary antibody (1:100 dilution; in blocking buffer) overnight at 4°C. After washing with PBT (6 times), embryos were post-fixed in 4% PFA and analyzed. For vascular branching analysis, the numbers of angiogenic vessels were counted from the 2D-image. For each genotype, five different embryos were scored and quantified.

### Histology and immunohistochemistry of embryos

Stage specific embryos were harvested from time-mated pregnant females. Embryos were fixed in 4% PFA overnight at 4°C and embedded. Histological sectioning was performed according to standard protocols [31]. Immunohistochemistry was performed on paraffin embedded sections (10 μm) using standard procedures [11, 24, 32]. Briefly, sections were rehydrated and heated in antigen retrieval solution (DAKO cytomation) for 20 min at 95°C, and blocked with 10% normal donkey serum at room temperature. Blocked sections were incubated overnight at 4°C with rat anti-endomucin (1:300, Abcam), mouse anti-CD31 (1:200, BD Bioscience) and chicken anti-GFP (1:400, Abcam). Sections were washed and incubated with Cy3- (1:500 dilution, Jackson ImmunoResearch Laboratories), and alexa488- (1:500, Abcam) conjugated secondary antibodies and were imaged using a Zeiss Axio Imager M1 upright microscope and AxioVision software. Vascular density of the transverse sections from 4–5 different embryos was quantified using ImageJ (1.47v) software. Embryo sections at three different levels for each embryo were examined and the vascular density was quantified.

### ES cell culture and ES/EB differentiation

Mouse embryonic stem (ES) cell culture and ES/EB differentiation was performed as described elsewhere [15, 16].The wild-type mouse E14 ES cell line was maintained in Knockout Medium (Invitrogen) supplemented with 15% FBS (Benchmark), 1000 U/ml LIF (Millipore), glutamine (Hyclone), 0.1 mM non-essential amino acids (NEAA) and 0.1 mM β-mercaptoethanol (Sigma) in gelatin-coated tissue-culture plates. The ES cells were differentiated into embryoid bodies (EBs) by the hanging drop method using mesodermal differentiation media containing IMDM (Invitrogen), 15% FBS, penicillin/streptomycin, 2 mM GlutaMAX, 50 mg/ml Fe-saturated transferrin (R & D Systems), 0.1 mM monothioglycerol (Sigma), and 50 mg/ml ascorbic acid (Sigma). Immunohistochemical analyses were performed using a standard protocol with anti-CD31 (1:200, BD Pharmingen) sera as previously described [29]. For EdU-incorporation assay, differentiating EBs was treated with EdU (10μM) for a period of 4h prior to harvest time point. The dissociated cells well fixed in 4% PFA for 10 min at room temperature, permeabilized and stained using Click-IT EdU kit (Thermofisher) for analysis.

### Flow cytometric analysis and sorting

FACS analysis was performed using BD FACSAria II (BD Biosciences, San Diego, CA, USA) as described previously [9, 17]. Stage specific embryos were separated from yolk sacs, and digested with 0.25% trypsin (Hyclone) to obtain a single cell suspension as previously described [11]. Cells were incubated with antibody cocktails for 30 minutes at 4°C, washed, and re-suspended in PBS with 2% FBS. Cocktails of antibodies CD31-APC (eBiosciences 25–0311), and VE-cadherin (BD Pharmigen) were used in this study, washed and re-suspended in FACS buffer (PBS/1%FBS). Cells were analyzed or sorted using FACSAria II (BD Biosciences). FACS data were quantified using data obtained from three independent experiments. AnnexinV-FITC labeling was performed according to the manufacturer’s protocol.

### RNA isolation and quantitative gene expression analysis

Total RNA was isolated from EBs at various time-points during ES/EB differentiation using miRVANA RNA isolation kit (Ambion), and cDNA was synthesized using superscript cDNA synthesis kit (Invitrogen) as per standard protocol. Quantitative RT-PCR was performed using ABI Taqman probe sets. Probes used include VIC labeled Gapdh: 4352339E, FAM labeled Timp2: Mm00441825_m1; Robo1: Mm00803879_m1; Robo2: Mm00620713_m1; Notch1: Mm00435245_m1; Hoxa5: Mm04213381_s1; Jarid2: Mm00445574_m1; Slit1:Mm01198620_m1; hsa-miR-130a:000454; U6 snRNA:01973.

### Matrigel-sandwich angiogenesis assay

For the angiogenesis assays, *miR-130a* iESCs were differentiated using a previously described protocol [29]. Doxycycline was added to the differentiating EBs from d2-d6 during differentiation. Uninduced and induced EBs were plated on growth factor reduced matrigel (BD Biosciences) containing 5% serum at day 8 of differentiation. Sprouting EBs were imaged at day 3 and day 6 following plating using an inverted microscope (Leica) supported with AxioVison (version 4.8). These assays were performed three times and in triplicate.

### Cell transfection and luciferase assays

HEK cells were transfected using Lipofectamine 3000 (Invitrogen) according to the manufacturer’s protocols. Briefly, for the *miR-130a* target assay, HEK cells were transfected using Lipofectamine 3000 (Invitrogen) with 2 μg of *pCMV-miR-130a* and 0.1 μg/well of *PGK-Luc-Jarid2-3’-UTR* and *PGK-Luc-Jarid2-3’-UTR* mutant constructs in 6-well plate. Transfected cells were harvested after 48 hrs and activities of *firefly* and *Renilla* luciferases were measured sequentially using the Dual-Luciferase Reporter Assay system (Promega) and a standard luminometer (Berthold Detection Systems, Sirius). Luciferase activities were expressed in relative light units that were normalized to the transfection efficiency using the *Renilla* luciferase activity.

### Zebrafish morpholino and *in situ hybridization* experiments

Zebrafish embryos were injected with 2.5 ng/embryo of mismatch control or *miR-130a* morpholinos at the one-two cell stage following standard protocols and approved by the UMN IACUC. Mismatch control or *miR-130a* morpholinos injected transgenic zebrafish *Tg(fli1a*:*EGFP)* embryos were analyzed at 48 hpf and 72 hpf. Perturbed ISV were quantified and plotted against total number of ISVs in the trunk region from control and *miR-130a* injected embryos. Quantification of the number of EGFP^+^ cells was performed using ImageJ software (NIH) and data obtained as mean intensity of the field reflecting the cell populations in each embryo. For FACS analysis, zebrafish embryos (n = 45) were dissociated using a buffer containing collagenase (1 mg/ml) for 5 min at 37°C. Dissociated cells were stained with propidium iodide (PI) and analyzed using FACS (FACSAria II, BD Biosciences). Embryos (n = 20) from each group were quantified and analyzed in triplicate. The following antisense MO oligonucleotides were designed by and obtained from the Gene Tools LLC as previously described [29]. *miR-130a*-MO: *5’-CAATGCCCTTTTAACATTGCACTGC-3’ miR-130a mis-MO-*1 (Control MO-1): *5’- CAATaCCaTTTTAAaATTaCACTaC-3’* (lower case represents mismatch bases) and *miR-130a mis-MO-*2 (Control MO-2): *5’- CAATGaCCaTTTAACAaTGaAaTGC-3’* (lower case represents mismatch bases). Whole mount *in situ hybridization* of 48 hpf zebrafish embryos was performed using standard procedures as described previously [33]. Zebrafish riboprobes included: *cdh5*, *kdrl* and *jarid2a*. For the zebrafish *in situ hybridization* experiments, 25–30 embryos were examined for each set. For the rescue experiments, *miR-130a* morpholinos together with negative control oligos or *miR-130a* mimics were injected at the one-two cell stage following standard protocols and analysed at 72hpf.

### Zebrafish RNA isolation and quantitative gene expression experiments

Zebrafish embryos were injected with 25 ng/embryo of mismatch control or *miR-130a* morpholinos at the one-two cell stage [33]. For total RNA isolation, a pool of 25–30 embryos were harvested in lysis buffer using miRVANA RNA isolation kit (Ambion), and cDNA was synthesized using the superscript cDNA synthesis kit (Invitrogen) as per standard protocol. qRT-PCR was performed for *jarid2a* using specific primers obtained from IDT. Primers specific for *gapdh* was used as a control.

### Tube formation assay

Tube formation assays were performed using HUVEC cells as described elsewhere [34]. Briefly, for tube formation assays, HUVECS were transfected with *Jarid2* constructs (0.25μg) using a nucleofector kit (Lonza), followed by plating 0.6 × 10^5^ cells on a 24-well plate coated with Low Growth Factor Matrigel (BD Biosciences, San Jose, CA) and supplemented with 50ng/ml of VEGF_165_ (R&D Systems, MN) in serum-free media. The tubes were imaged at 10X magnification and quantified from 4 different fields. Co-transfection of *Jarid2* and *miR-130a* (100nM) was performed as described above.

### *miRNA* pull down assay

A biochemical approach was used to confirm the interaction of *miR-130a* with *Jarid2* mRNA as described elsewhere [35]. Briefly, 3’-biotinylated miRCURY LNA *miR-130a-3p* mimics (cat# 479997–671, design id #713696) and a negative control (cat# 479997–671, design id #713697) were purchased from Exiqon. Mouse ES cells were transfected with 30 pmol of the *miRNA* mimics using RNAiMAX reagent (cat# 13778–030, Thermo Fisher). After 24 hours of transfection, cells were harvested and cytosolic extracts were obtained in lysis buffer. mRNAs bound to the biotinylated-miRNAs were then pulled down using Streptavidin-Dynabeads (cat# 11205D, Thermo Fisher). The beads were washed and treated with RNase-free DNase to remove any residual DNA. The pulled down RNA was eluted and cDNA was sysnthesized using the SuperScript™ III First-Strand Synthesis SuperMix (cat# 18080400, Thermo Fisher). The abundance of *Jarid2* mRNA in the pulldown samples was assessed using qPCR and normalized to the levels of Gapdh as a negative control.

### RNA-electrophoretic mobility shift assay (EMSA)

RNA-EMSAs were performed as described elsewhere [36]. Briefly, RNA oligos were ordered corresponding to the mature form of *miR-130a-3p* (*5’-CAGUGCAAUGUUAAAAGGGCAU-3’*), a 21-mer sequence of the *Jarid2 3’-UTR* (*5’-Auagcuacccacauugcacug-3’*) containing the target site for *miR-130a* and a scrambled control (*5’-GGuuAACuCGCCAAuGAuCCu-3’*) from IDT. The *miR-130a-3p* sequence was labeled with 5’-IRDye 700. Labeled *miR-130a-3p* probes (200 nM) were incubated with the corresponding target or scrambled RNA molecules in the presence of 10 mM MgCl_2_, 100 mM NaCl, 50 mM Hepes pH 7.2 and 5% glycerol for 30 minutes at 37°C. Binding reactions were run in a 12% polyacrylamide gel for 3 h at 120 V (4° C) in 1X TBE. Gels were scanned using a LI-COR Odyssey CLx system and assembled.

### Statistical analysis

All experiments were repeated at least three times and the data represent the mean ± SEM. Images were analyzed using ImageJ software (NIH). Statistical significance was determined using the Student’s *t*-test and differences are considered significant with a p-value of < 0.05 and very significant; p < 0.01.

## Results

### *Etv2-Cre*-mediated *Dicer* deletion results in vascular patterning defects

**Fig 1:**
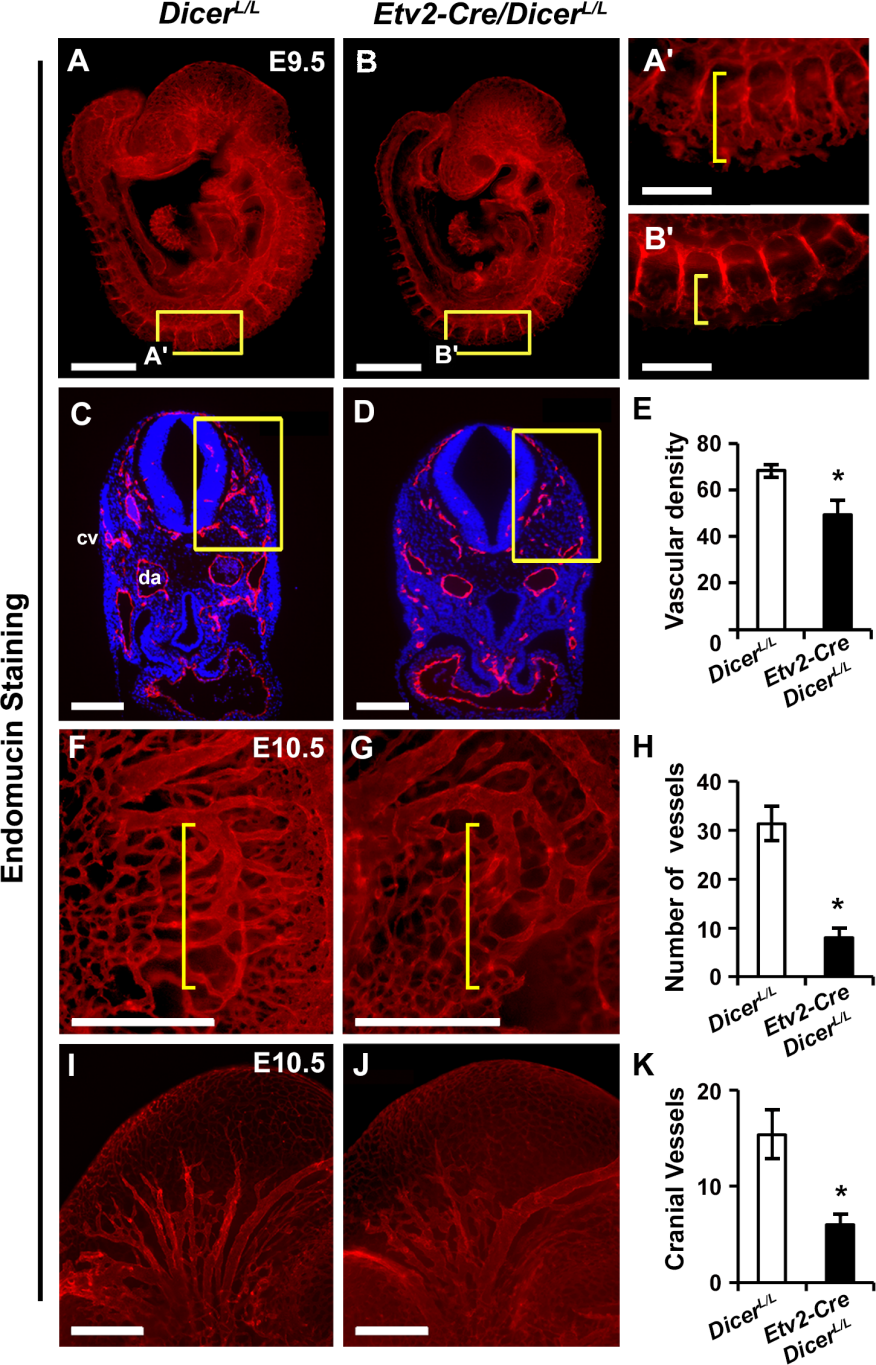
*Etv2-Cre*-mediated *Dicer* deletion results in vascular abnormalities. **A-D**, Representative images of whole-mount embryos (A, B) and transverse sections (C, D) labeled with endomucin antibody (red) and counterstained with DAPI (blue) in the developing embryos at E9.5. Higher magnification (A’, B”) of the boxed region in panel A and B. **E**, Quantification of the boxed areas in panel C and D. **F-K**, Whole-mount endomucin labeled (red) images of wild-type (F, I) and mutant (G, J) embryos at E10.5 in the trunk (F, G [yellow brackets]) and middle cranial region (I, J). **H, K**, Quantitative analyses of vascular branching and cranial vessels at E10.5. Error bars indicate SEM (n = 4; *p < 0.05). Scale bar: 500 μm (A, B, F, G, I and J); 200 μm (A’, B’, C and D).

*Etv2* (Ets-family transcription factor) marks the earliest hematopoietic and endothelial progenitors [9, 20]. Homozygous global deletion of *Etv2* results in complete loss of hematopoietic and endothelial lineage and embryonic lethality [8, 9]. We previously generated *Etv2*^*Cre/+*^;*Dicer*^*L/L*^ progeny and observed that these conditional mutant embryos were lethal by E12.5 [29]. Therefore, our initial analysis focused on the lethality of the *Etv2*^*Cre/+*^;*Dicer*^*L/L*^ embryos. Analysis of the stage specific *Dicer*^*L/L*^ and *Etv2*^*Cre/+*^;*Dicer*^*L/L*^ embryos were indistinguishable at E8.0, however, we found significantly reduced size at E9.5 as compared to the control littermates (S1A and S1B Fig). We next performed immunohistochemical analysis using endomucin antibodies at two developmental stages, E9.5 and E10.5, to visualize the endothelial development in *Dicer*^*L/L*^ and *Etv2*^*Cre/+*^;*Dicer*^*L/L*^ embryos. Immunohistochemical analysis of E9.5 embryos (whole mount imaging as well as tissue sections) revealed the presence of major vessels including the dorsal aorta (da) and cardinal vein (cv) in both *Dicer*^*L/L*^ and *Etv2*^*Cre/+*^;*Dicer*^*L/L*^ embryos (Fig 1A–1D). Although the initial vascular structures were present in the mutant embryos, higher magnification revealed poorly developed vascular plexuses with reduced vascular branching in *Etv2*^*Cre/+*^;*Dicer*^*L/L*^ as compared to *Dicer*^*L/L*^ embryos (Fig 1A’ and 1B’). Reduced plexus formation was also evident in the transverse sections of E9.5 embryos with fewer endomucin-positive vessels in the mutant embryos (Fig 1C and 1D). Quantitative analysis within the paraxial mesoderm revealed significantly reduced vascular density in the *Etv2*^*Cre/+*^;*Dicer*^*L/L*^ embryos relative to the *Dicer*^*L/L*^ control littermates (Fig 1E). To verify these results, we undertook immunohistochemical analysis using anti-CD31 antibodies using *Dicer*^*L/L*^ and *Etv2*^*Cre/+*^;*Dicer*^*L/L*^ embryo sections at E9.5. Consistent with the endomucin staining, we observed reduced levels of CD31 staining in the *Etv2*^*Cre/+*^;*Dicer*^*L/L*^ sections as compared *Dicer*^*L/L*^ sections (S1C, S1C’, S1D and S1D’ Fig). To further validate these results, we crossed *Etv2*^*Cre/+*^;*Dicer*^*L/+*^ *Etv2-EYFP*;*Dicer*^*L/L*^ lines and performed immunohistochemical analysis using anti-CD31 and anti-GFP antibodies at the somite level to visualize inter-somitic vessels. Our analysis revealed a marked reduction in the expression of both EYFP and CD31 in these regions of *Etv2*^*Cre/+*^;*Dicer*^*L/L*^ embryos as compared to *Dicer*^*L/L*^ embryos (S1E–S1H Fig). By E10.5, we observed a reduced number of vessels in the trunk-region and perturbed branching of cranial vessels in the *Etv2*^*Cre/+*^;*Dicer*^*L/L*^ embryos (Fig 1F–1K).

**Fig 2:**
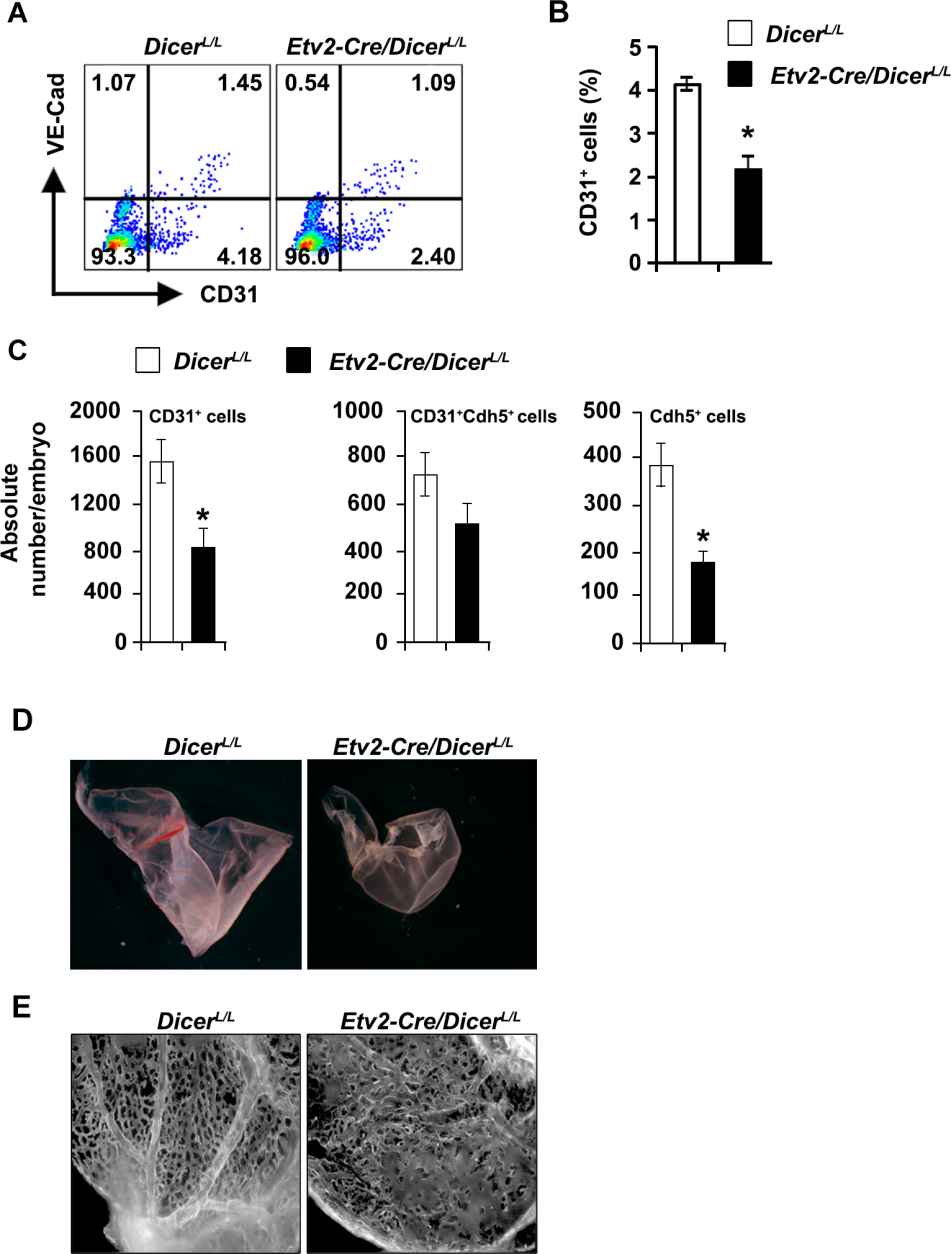
*Etv2-Cre*-mediated *Dicer* deletion results in vascular abnormalities. **A**, FACS profile of endothelial populations (VE-cadherin and CD31) from *Dicer*^*L/L*^ and *Etv2*^*Cre/+*^*;Dicer*^*L/L*^ embryos at E10.5. **B, C,** Quantitative analysis of endothelial markers from *Dicer*^*L/L*^ and *Etv2*^*Cre/+*^*;Dicer*^*L/L*^ embryos. Note the reduced percentage and absolute number of endothelial populations in Cre^+^ embryos. **D,** Whole-mount imaging of yolk-sac from *Dicer*^*L/L*^ and *Etv2*^*Cre/+*^*;Dicer*^*L/L*^ yolk-sac. The absence of defined blood vessels were apparent in the *Etv2*^*Cre/+*^*;Dicer*^*L/L*^ yolk-sac. **E,** Whole-mount endomucin labeled images of wild-type and mutant yolk-sac at E10.5. Note the defective vasculature in the yolk-sac of *Etv2*^*Cre/+*^*;Dicer*^*L/L*^ embryos. Error bars indicate SEM (n = 3; *p < 0.05).

To examine whether the vascular defects were due to altered endothelial lineages, we undertook FACS analysis to assess the endothelial cell populations in the *Etv2*^*Cre/+*^;*Dicer*^*L/L*^ and *Dicer*^*L/L*^ embryos. FACS analyses at E10.5 using CD31 and VE-cadherin antibodies showed significantly reduced number of endothelial cell populations in the *Etv2*^*Cre/+*^;*Dicer*^*L/L*^ embryos compared to the wild-type littermates (Fig 2A–2C). We next evaluated vascular patterning in the developing yolk-sac to further characterize these *Etv2*^*Cre/+*^;*Dicer*^*L/L*^ embryos. Similar to the embryo proper, the whole-mount imaging of the yolk-sac showed perturbed vascular development and were pale colored consistent with decreased blood content (Fig 2D). Further, whole-mount endomucin immunohistochemistry of the yolk sac revealed poorly developed vascular plexuses in the mutants (Fig 2E).

These observations suggested that the loss of *Dicer* in the endothelial precursors resulted in defective vascular remodeling. Overall, these results supported the hypothesis that Dicer and *miRNAs* were required for angiogenesis during development.

### *miR-130a* regulates angiogenesis *in vitro*

**Fig 3:**
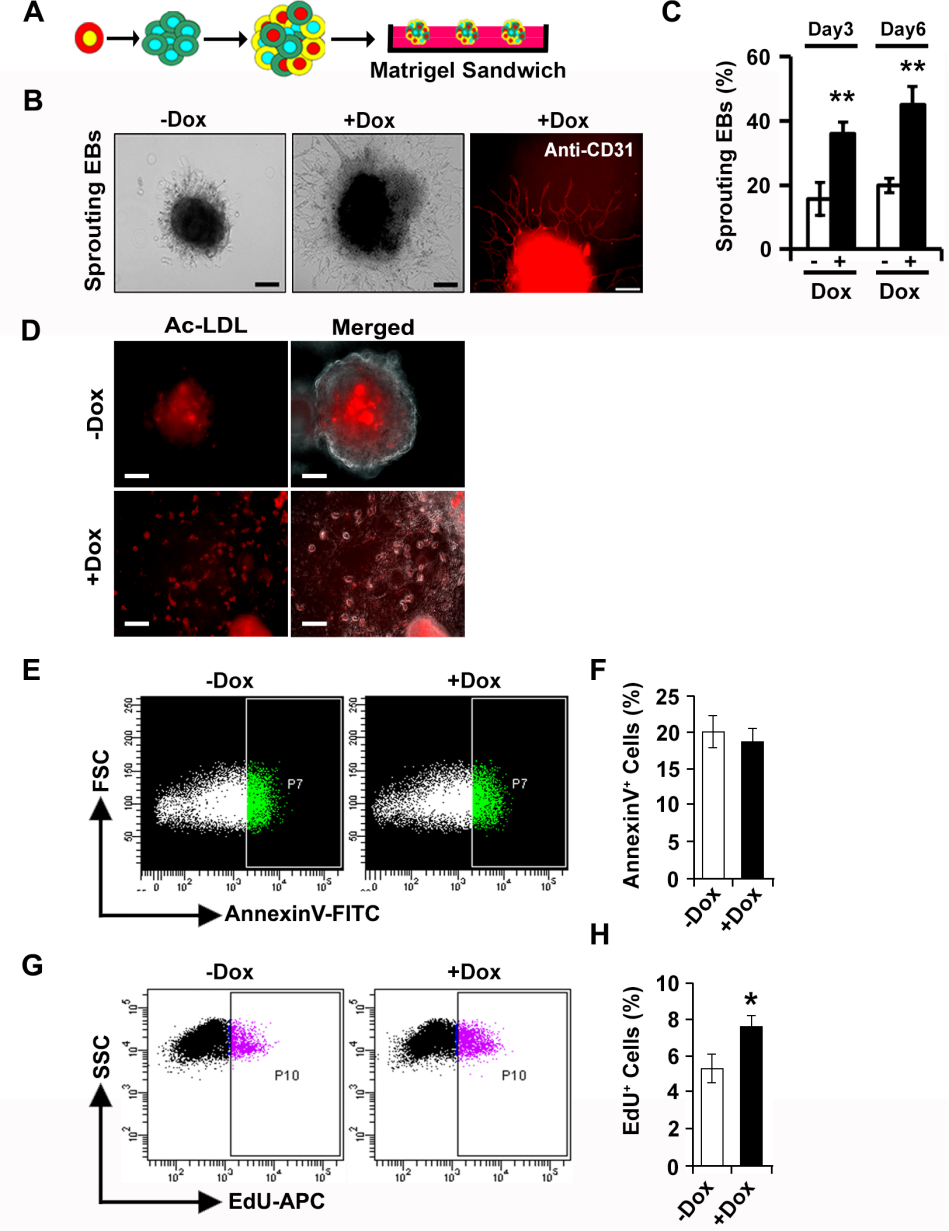
*miR-130a* regulates angiogenic response *in vitro*. **A,** Schematic showing the matrigel sandwich angiogenesis assay. **B,** Representative images of sprouting EBs from -Dox and +Dox conditions 6-days after plating are shown. Extensive endothelial [CD31 (red)] sprouting was observed in +Dox condition. **C,** Quantification of sprouting EBs at day 3 and day 6 of plating revealed significantly increased percentage of sprout formation upon induction of *miR-130a*. **D,** Ac-LDL uptake assay in EBs differentiated in the absence and presence of Dox. The cells migrating out of EBs incorporated Ac-LDL (red), indicating enhanced angiogenic response upon induction of *miR-130a*. Merged panel shows overlay with phase contrast images. **E, F,** FACS analysis and quantification of Annexin V-FITC labeled cells in the absence (-Dox) and presence (+Dox) of doxycycline. **G, H** FACS analysis and quantification of EdU labeled cells in the absence (-Dox) and presence (+Dox) of doxycycline. Error bars indicate SEM (n = 4; *p < 0.05, **p < 0.01).

Previously, we identified *miR-130a* as an endothelial enriched *miRNA* and established its role in endothelial specification [29]. To further examine the role of *miR-130a* during the endothelial development, we first utilized the Dox-inducible mouse ES cell line to over-express *miR-130a* and determined whether *miR-130a* could modulate angiogenic functions using a matrigel sandwich sprouting assay. Uninduced and induced *miR-130a* iES/EBs at d6 were plated on a growth factor-reduced Matrigel sandwich supplemented with VEGF (50ng/ml) and 5% serum medium (Fig 3A). Dox-mediated induction of *miR-130a* resulted in a robust increase of EBs with angiogenic sprouts (35–40%) relative to the uninduced EBs (12–15%) [Fig 3B and 3C]. We next performed immunohistochemical analysis to determine whether the sprouts were endothelial populations. Our immunohistochemical analysis using CD31 antibodies confirmed the presence of endothelial cells in the sprouting EBs (Fig 3B). We then quantified the sprouting EBs at two different time points, d3 and d6, of plating to evaluate the role of *miR-130a* in the angiogenic response. Quantitative analysis revealed extensive sprout formation both at d3 (3-fold) and d6 (2.5-fold) in the presence of Dox (+Dox) as compared to the controls (-Dox) (Fig 3C). To determine whether these angiogenic sprouts consisted of functionally active endothelial cells, we performed an acetylated low-density lipoprotein (Ac-LDL) incorporation assay. Our fluorescence microscopic analysis revealed Ac-LDL updake by the migrating endothelial cells, further confirming the endothelial nature of these angiogenic sprouts (Fig 3D). These results confirmed the role of *miR-130a* in angiogenesis. To evaluate whether the increased sprouting was due to the prevention of cell death, we performed a cell death assay using Annexin V staining using the differentiating EBs. FACS analysis of d6 uninduced and induced EBs revealed no significant differences in the percentage of Annexin V staining (Fig 3E and 3F). Furthermore, flourescence microscopy did not show any difference in the number of Annexin V stained cells (S2 Fig). We then monitored the proliferative response following induction of *miR-130a* in the differentiating EBs. FACS analysis using a ClickIT-EdU assay kit revealed that Dox-mediated induction of *miR-130a* resulted in a modest but significant increase in the percent of EdU^+^ cells as compared to uninduced EBs (Fig 3G and 3H).

Overall, these results indicated that the increased angiogenesis and sprouting following the induction of *miR-130a* was not solely due to the prevention of cell death or enhanced cellular proliferation.

### *miR-130a* regulates endothelial patterning *in vivo*

**Fig 4:**
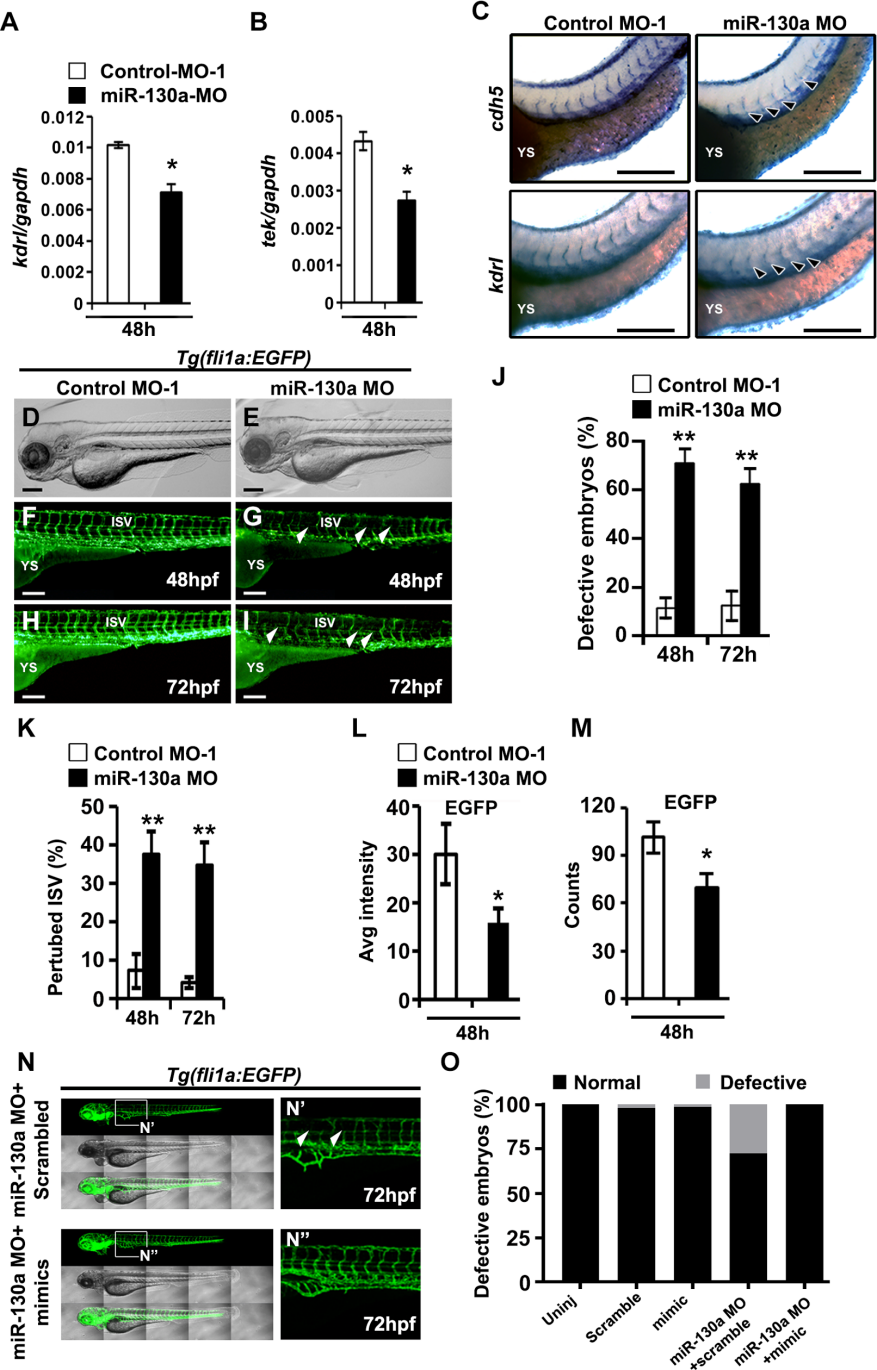
*miR-130a* regulates endothelial patterning *in vivo*. **A, B,** qPCR analysis of endothelial transcripts, *kdr1* and *tek* at 48hpf using RNA from control and *miR-130a* morphants. **C,** Whole-mount *in situ* hybridization images of control and *miR-130a* morphants at 48 hpf using *cdh5* and *kdr1* probes. Note the defective nature of vascular development and reduced expression of these transcripts in *miR-130a* morphants (arrowheads). **D, E,** Brightfield microscopic images revealed no major changes in gross morphology of mismatch control and *miR-130a* morphants. **F-I,** Lateral fluorescence images of *Tg(fli1a*:*EGFP)* zebrafish lines revealed defective vasculature in *miR-130a* morphants (white arrowheads) at 48 hpf (F, G) and 72 hpf (H, I) time periods. **J, K,** Quantitative analysis of the number of defective zebrafish embryos with perturbed inter-somitic vessels (ISVs) at 48 hpf and 72 hpf. **L, M,** ImageJ (L) and FACS (M) analyses of EGFP^+^ cells revealed significantly reduced EGFP intensity and counts in *miR-130a* morphants. **N,** Lateral fluorescence and brightfield images of Tg(*fli1a*:*EGFP*) zebrafish lines co-injected with *miR-130a* morpholinos and LNA modified scrambled oligos and *miR-130a* mimics at 72hpf. Panel N’ and N” shows the enlarged images of the boxed area in panel N. Note the restoration of the vascular structures following co-injection with *miR-130a* morpholinos and *miR-130a* mimics (N’, N”; arrowheads). **O,** Quantitative analysis of the number of defective zebrafish embryos with perturbed inter-somitic vessels (ISVs) 72 hpf. Error bars indicate SEM (n = 3; *p<0.05; **p < 0.01). Scale bar: 200 μm.

Having established the role of *miR-130a* in promoting endothelial differentiation and angiogenic response *in vitro*, we assessed the *in vivo* function of *miR-130a* using morpholino (MO) oligonucleotide-mediated knockdown experiments in zebrafish embryos. We designed specific MOs and injected control mismatch and *miR-130a* MOs (25 ng/embryo) at the 1–2 cell stage and first performed qPCR analysis using RNA isolated from 60-pooled embryos from each condition for the endothelial transcripts at 48hpf. As compared to control morphants, qPCR experiments revealed significantly reduced expression of *kdrl* and *tek* in *miR-130a* morphants (Fig 4A and 4B). To validate these results, we performed *in situ* hybridization experiments using *kdrl* and *cdh5* probes in the control and *miR-130a* morphants at 48 hpf. *In situ hybridization* experiments for *kdrl* and *cdh5* showed perturbed vasculature with reduced expression of both *kdrl* and *cdh5* in the inter-somitic vessels (ISVs) in the *miR-130a* morphants (Fig 4C). These results indicated an important role of *miR-130a* in vascular patterning *in vivo*. To further evaluate its function during the endothelial patterning, we utilized the *fli1a*:*EGFP* transgenic reporter line [*Tg(fli1a*:*EGFP*], which expresses GFP in the developing vasculature [37]. As compared to control morphants, the injection of *miR-130a* MO did not cause detectable change in gross morphology of the developing embryos (Fig 4D and 4E). We did not observe any defects in the developing vasculature in the control morphants (Fig 4F and 4H). Although gross morphology of the developing embryos was not significantly changed (Fig 4D and 4E), we found multiple defective embryos with perturbed vasculature (Fig 4F–4J). Additionally, we found incompletely formed inter-somitic vessels (ISVs) in the *miR-130a* MO injected embryos at both 48 hpf and 72 hpf (Fig 4F–4K). These defects were observed in 70% ± 5% and 60% ± 7% (n = 30 embryos per experiment repeated three times) of the *miR-130a* MO injected embryos at 48 hpf and 72 hpf, respectively (Fig 4J and 4K). To evaluate the specificity of the injected MO, we designed a second control MO (control-MO-2) and performed similar experiments and observed the same results as the control-MO-1 injections (S3A–S3G Fig). These findings indicated the critical role of *miR-130a* in angiogenesis, vascular remodeling and sprouting process during development. Quantitative analysis of EGFP^+^ using ImageJ software (NIH) revealed significantly reduced mean EGFP intensity in the *miR-130a* morphants as compared to control morphants (Fig 4L). Next, we performed FACS analysis to quantify the EGFP^+^ endothelial populations following injection with control and *miR-130a* morpholinos at 48hpf. As observed with the qPCR and ImageJ analyses, FACS profiling of the *Tg(fli1a*:*EGFP)* zebrafish embryos revealed significantly reduced number of EGFP counts in the *miR-130a* morphants as compared to the control morphants (Fig 4M). To further demonstrate the role of *miR-130a* during vascular development, we performed rescue experiments using *Tg(fli1a*:*EGFP)* transgenic lines. We injected LNA-modified scrambled oligos and *miR-130a* mimics together with *miR-130a* morpholino at the 1–2 cell stage. Confocal microscopic analysis at 72hpf revealed that co-injection of *miR-130a* morpholino with scrambled oligos resulted in perturbed vasculature. These defects were rescued by co-injecting *miR-130a* mopholinos and *miR-130a* mimics (Fig 4N and 4O). Quantitative analysis at 72hpf showed 30%± 2% (n = 70 embryos) embryos with severely defective vasculature in the *miR-130a* morpholino with scrambled mimic groups whereas, none were found to have defects in the *miR-130a* morpholino with *miR-130a* mimic groups (Fig 4O).

Overall, these *in vivo* experiments demonstrated the critical role of *miR-130a* in vascular remodeling and sprouting process during embryonic development.

### *miR-130a* targets *Jarid2* expression during development

**Fig 5:**
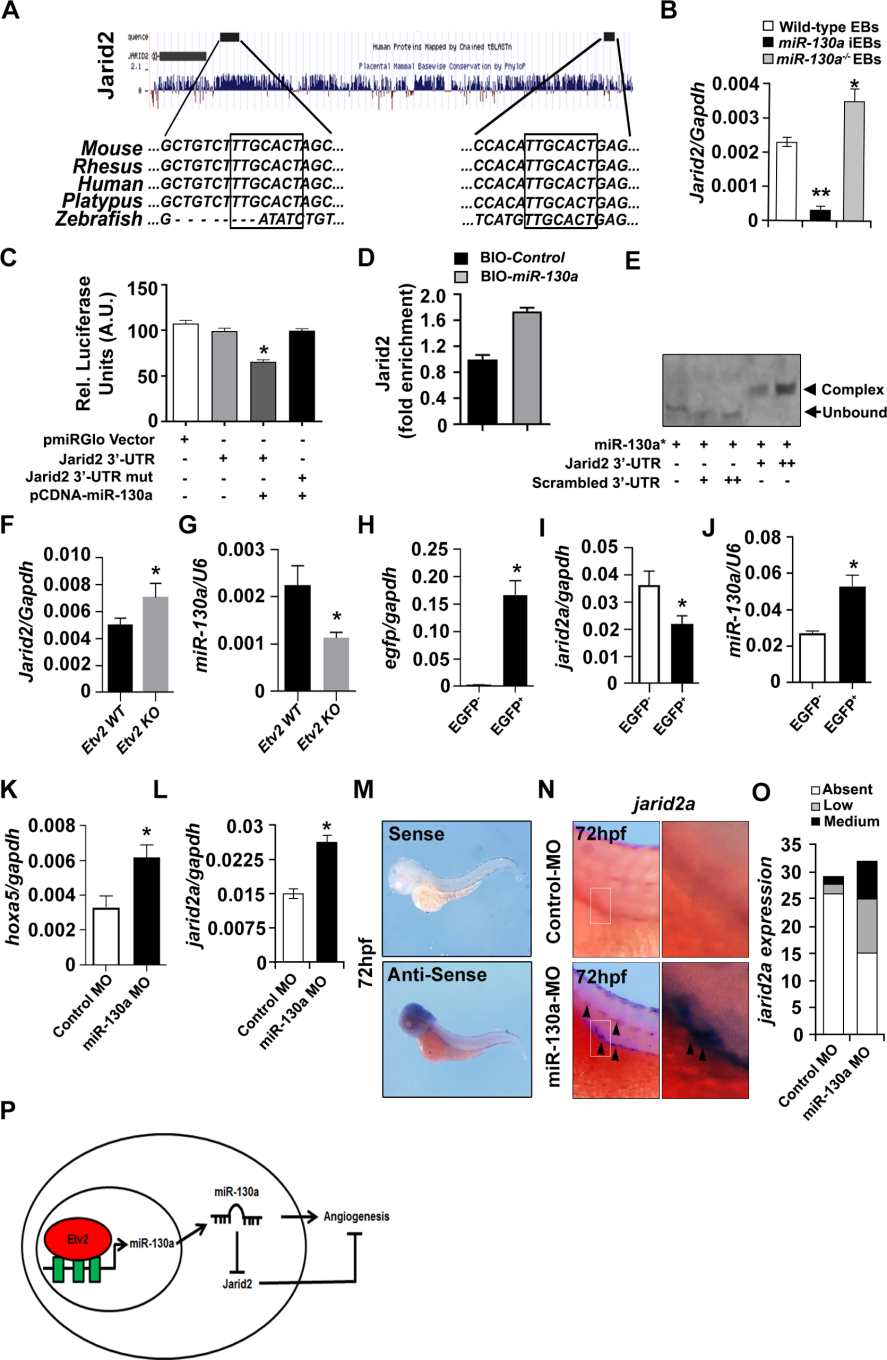
*miR-130a* targets *Jarid2* in the developing vasculature. **A,** MultiAllign sequence alignment of *Jarid2 3’ UTR* showing conservation across different vertebrates with a conserved *miR-130a* binding motif in the 3’-UTR region. **B,** qPCR analysis of *Jarid2a* expression using RNA isolated from wild-type EBs, *miR-130a* iEBs and *miR-130a-null* EBs at d6 of differentiation. **C,** Luciferase activity of PGK-*Luc-Jarid2-3’-UTR* and PGK-*Luc-Jarid2-3’-UTR* mutant reporter constructs in the presence of pCMV-*miR-130a* constructs. **D,** *miRNA* pull down assay using biotinylated-*miR-130a* showing ~1.75-fold enrichment of *Jarid2 3’-UTR* in the *miR-130a* transfected cells as compared to scrambled control. **E,** RNA-EMSA assay using 5’-IRDye700-labeled *miR-130a* (miR-130a*) to show direct binding of *miR-130a* and *Jarid2 3’-UTR* region. Note the presence of a complex formation upon co-incubation of *Jarid2 3’-UTR* oligo with *miR-130a* resulted, but not in the scrambled oligo. **F, G,** qPCR analysis of *Jarid2a* and *miR-130a* transcripts using RNA isolated from age-matched wild-type control and *Etv2-*null mouse embryos at E8.5. **H-J,** qPCR analysis of *egfp*, *jarid2a* and *miR-130a* transcripts at 72hpf using RNA isolated from EGFP^-^ and EGFP^+^ sorted cells obtained from *Tg(fli1a*:*EGFP)* transgenic lines. **K, L,** qPCR analysis of *hoxa5a* and *jarid2a* transcripts at 72hpf using RNA from control and *miR-130a* morphants. **M,** Whole-mount *in situ* hybridization images of the wild-type embryos using *jarid2a* sense and anti-sense probes at 72hpf. **N,** Whole-mount *in situ* hybridization images of control and *miR-130a* morphants at 72 hpf using *jarid2a* probes. Note the enhanced expression of *jarid2a* in the developing vasculature (arrowheads). **O,** Quantitative analysis of the number of zebrafish embryos with increased expression of *jarid2a*. **P,** Schematic showing the regulation of the *Etv2-miR-130a-Jarid2* cascade in the angiogenic pathway. Error bars indicate SEM (n = 3; from 20 pooled embryos; *p<0.05; **p < 0.01).

*miRNAs* can regulate the expression of multiple genes by binding to the *3’-UTR* of the target genes [25]. To decipher the mechanism by which *miR-130a* regulates embryonic vascular development, we utilized three *miRNA* target prediction tools including TargetScan 6.2, PicTar and miRANDA to mine common predicted targets between mouse and zebrafish genomes. Among the several targets, we identified *Jarid2* as a top-ranked candidate with a high percentile score using TargetScan 6.2 in both mouse and zebrafish genomes (Fig 5A and S1 Table). To validate these predicted targets, we utilized wild-type *miR-130a* inducible and CRISPR/Cas9-mediated *miR-130a* knockout ES/EB system and performed qPCR analysis at d6 of differentiation. Besides *Jarid2*, we also evaluated the expression of multiple *miR-130a* predicted targets including *HoxA5*, *Robo-Slit* signaling, *Timp2* and *Notch*, that are shown to have a role in endothelial patterning using RNA isolated from wild-type EBs, *miR-130a* iEBs and *miR-130a*^*-/-*^ d6 EBs (S4A–S4F Fig). As expected the levels of *HoxA5* (a known target of *miR-130a* [38]) was decreased upon Dox-mediated induction of *miR-130a* (S4A Fig). Accordingly, the levels of *HoxA5* were increased in the *miR-130a-null* EBs relative to the wild-type EBs (S4A Fig). These results supported the previously defined role of *miR-130a* in the regulation of anti-angiogenic molecules (HoxA5) [38]. We found a similar expression pattern for *Jarid2*, *Robo1* and *Robo2* transcripts, whereas the expression of *Slit1*, *Timp2* and *Notch1* did not show any such trend in the wild-type EBs, *miR-130a* iEBs and *miR-130a*^*-/-*^ EBs (Fig 5B and S4B–S4F Fig). Based on the bioinformatics analysis and qPCR results, we hypothesized that *miR-130a* target *Jarid2*, *Robo1* and *Robo2* transcripts by binding to the *3’ UTR* of these genes in the regulation of endothelial patterning. An earlier study has demonstrated the role of *miR-218* in vascular patterning by modulating *Robo-Slit* signaling [39]. Therefore, we focused on *Jarid2* expression, as little is known about its role in vascular patterning and its *miRNA*-mediated regulation during development. Sequence alignment using Multiz align (UCSC genome browser/mm9) revealed two highly conserved *miR-130a* seed-sequences in the *Jarid2*-*3’-UTR* region (Fig 5A). To decipher whether *miR-130a* could bind to the *Jarid2*-*3’-UTR* region and regulate its expression, we performed luciferase assays using a reporter construct harboring the *miR-130a* binding motif of the *Jarid2-3’-UTR* region (*PGK-Luc-Jarid2-3’ UTR*). Co-transfection of HEK cells with *PGK-Luc-Jarid2-3’ UTR* and *pCMV-miR-130a* construct led to a robust and significant reduction (~35%) in luciferase activity. In contrast, co-transfection with mutated *Jarid2 3’-UTR* (*Jarid2-3’ UTR mut)*, *the pCMV-miR-130a* construct resulted in unaltered luciferase activity (Fig 5C). Next, we performed *miRNA* pull down assay using biotinylated-*miR-130a* and demonstrated an interaction between *miR-130a* and *Jarid2* mRNA. Our data showed ~1.75-fold enrichment of *Jarid2 3’-UTR* in the *miR-130a* transfected cells as compared to the scrambled control (Fig 5D). To further validate these results, we performed in vitro RNA-EMSA to demonstrate direct binding of *miR-130a* and *Jarid2 3’-UTR* region. We found that co-incubation of *Jarid2 3’-UTR* oligo with *miR-130a* resulted in a complex formation, whereas, the scrambled oligo did not show any mobility shift (Fig 5E). These results indicated that *miR-130a* directly binds to *Jarid2* mRNA and regulates its expression.

Previously, we have demonstrated Etv2 as an upstream regulator of *miR-130a* [29]. Etv2 marks the earliest endothelial precursors [9]. To monitor the expression of *Jarid2* in the angioblasts and endothelial cells, we sorted EGFP^-^ and EGFP^+^ cells using *Etv2-EYFP* transgenic wild-type mouse embryos at E8.5 and performed qPCR analysis. Our data showed that *Jarid2* transcripts were expressed in both EGFP^-^ as well as EGFP^+^ cells, however, the levels of *Jarid2* was lower in the EGFP^+^ cells as compared to EGFP^-^ cells (S5A–S5D Fig). Next, we investigated whether the expression of *Jarid2* was altered in the *Etv2*-null embryos during development. Our qPCR analysis using RNA isolated from the wild-type and *Etv2*^*-/-*^ embryos revealed significantly increased expression of *Jarid2* mRNAs in the *Etv2*^*-/-*^ embryos as compared to the age-matched wild-type embryos (Fig 5F). In order to determine which cell populations have increased levels of *Jarid2* mRNA in the *Etv2*^*-/-*^ embryos, we analysed the microarray datasets obtained from EGFP^-^ and EGFP^+^ sorted cells using wild-type and *Etv2*^*-/-*^ embryos at E8.5 [11]. We did not find any differences in the levels of *Jarid2* mRNA within the EYFP^-^ population; however, the levels of *Jarid2* transcripts were increased in the EYFP^+^ populations in the Etv2^-/-^ embryos as compared to the wild-type embryos (S5E Fig). To decipher whether *miR-130a* expression was altered in the *Etv2*^*-/-*^ embryos, we performed qPCR experiments for *miR-130a* using RNA isolated from the wild-type and *Etv2*^*-/-*^ embryos. Our analysis indicated that the level of *miR-130a* was decreased in the *Etv2*^*-/-*^ embryos as compared to wild-type littermates (Fig 5G). Based on the reciprocal expression of *miR-130a* and *Jarid2* in the wild-type vs. *Etv2*^*-/-*^ embryos, we propose that the levels of *Jarid2* are modulated by *miR-130a* in the angiogenic response. To test this hypothesis, we utilized HUVEC cells and performed tube formation assays following the over-expression of *Jarid2*. We observed extensive tube formation in the vector transfected cells, whereas, over-expression of Jarid2 resulted in defective tube formation. These results suggested an inhibitory role of Jarid2 during vascular development (S6A and S6B Fig). We then co-expressed *Jarid2* and *miR-130a* mimics to examine whether *miR-130a* could rescue the defective tube formation phenotype. Our data revealed that expression of *Jarid2* together with *miR-130a* mimics resulted in significant restoration of the tube formation (S6C Fig). Collectively, these results supported the notion that *Jarid2* and *miR-130a* interacted and impacted vascular development.

Next, we examined whether *miR-130a* could regulate the expression of *jarid2a* transcripts *in vivo*. Initially, we designed *jarid2a* qPCR probes and examined the expression of *jarid2a* in the endothelial populations of the developing zebrafish embryos. qPCR analysis using RNA isolated from the sorted EGFP^-^ and EGFP^+^ cells at 72hpf revealed that *jarid2a* was expressed in both EGFP^-^ as well as EGFP^+^ cells, however, at lower levels in the EGFP^+^ cells relative to EGFP^-^ cells (Fig 5H and 5I). We next evaluated the expression of *miR-130a* in these sorted cell populations. We found that comparable to the mouse endothelium, *miR-130a* was highly enriched in the EGFP^+^ cells as compared to EGFP^-^ cells (Fig 5J). These results supported the notion that *miR-130a* plays an important role in the regulation of *jarid2a* in the developing vasculature. To analyse this hypothesis, we performed qPCR analysis using RNA isolated from control and *miR-130a* morphants at 72hpf. qPCR analysis for *hoxa5a* transcripts (a known target of miR-130a [38]) showed enrichment of *hoxa5a* mRNAs in the *miR-130a* morphants relative to control morphants, indicating a functional repression of *miR-130a* in the injected morphants (Fig 5K). Next, our qPCR analysis revealed significant enrichment of *jarid2a* transcripts in the *miR-130a* morphants relative to the control morphants (Fig 5L). To validate these results, we injected control and *miR-130a* morpholinos at the one-cell stage and performed *in situ hybridization* using *jarid2a* probes at 72hpf of the developing zebrafish embryos (Fig 5M). We found no detectable expression of *jarid2a* transcripts within the developing vasculature in the control embryos, whereas, injection of *miR-130a* morpholinos showed an increased expression of *jarid2a* in the *miR-130a* morphants within the developing vasculature (Fig 5N). Quantitative analysis further revealed an increased number of the embryos with enriched *jarid2a* expression in the *miR-130a* morphants relative to the controls (Fig 5O).

These results support the hypothesis that *miR-130a* regulates *jarid2* transcripts both *in vitro* and *in vivo*, thereby regulating vascular patterning.

## Discussion

During vascular development, coordinated endothelial cell behavior is critical for functional blood vessel formation [1, 2, 22]. Deciphering the transcriptional and post-translational mechanisms that fine-tune key regulatory network to control vasculogenesis and angiogenesis is critical for embryogenesis. Several *miRNAs* have been shown to play an essential role by regulating the expression of multiple components of complex biological pathways [25, 26]. Here, we have uncovered an essential role of miRNAs in endothelial patterning. Specifically, we showed the importance of *miR-130a* in the vascular and angiogenic response during embryonic development. Mechanistically, *miR-130a* binds to the 3’ UTR of *Jarid2* and modulates transcript expression and vascular patterning during embryogenesis.

*miRNAs* (small non-coding RNAs) biogenesis and their maturation is mediated by *Dicer* (a RNA binding protein) [25, 27]. Global as well as hypomorphic mutants of *Dicer* are embryonic lethal, however, only a few studies have described the functional role of *Dicer* in a lineage specific fashion [27, 28]. Fox example, *Nkx2*.*5-Cre* mediated deletion of *Dicer* resulted in embryonic lethality [40], whereas, the deletion by *Tie2-* or *VE-Cadherin-Cre* produced viable progeny [41]. In our previous study, we demonstrated that *Dicer* and *miRNA* function in the endothelial progenitors (angioblasts) were essential for vascular development [29]. The present study further extends the role of *miRNAs* in the later stages of vascular development in the regulation of endothelial patterning. Since *Tie2-* or *VE-Cadherin-Cre* mediated *Dicer-floxed* deletion resulted in a viable phenotype whereas Etv2-Cre-mediated *Dicer-floxed* deletion led to embryonic lethality [29], we hypothesize that *miRNAs* expressed at an earlier stage are essential for endothelial precursors and survival. It is also possible that during the late stages of embryonic development, *Dicer* functions are not essential for survival; however it is required for the vascular integrity to facilitate the process of angiogenesis and vascular patterning. These findings suggest developmental stage and lineage specific role of *miRNAs* during embryogenesis.

A previous study from our laboratory identified an *Etv2-miR-130a-Pdgfra* cascade in the regulation of mesodermal specification [29]. In this study, we report yet another function of *miR-130a* in the regulation of vascular patterning and angiogenesis both *in vitro* and *in vivo*. Several other *miRNA* clusters including *miR-126*, *miR-218*, *miR-23/27* clusters are documented in the regulation of angiogenesis [39, 42–44], whether, all these families of *miRNAs* function independently or in concert are unclear. Many other growth factors including VEGF, FGF, PDGFR-B are known to promote angiogenesis and regeneration [21, 22]. It is possible that *miRNAs* including *miR-130a* modulate the angiogenic response mediated via interacting with these signaling cascades. Therapeutically, *miR-130a*-mediated induction of neoangiogenesis might be useful in pathophysiological conditions such as ischemic heart disease, whereas, specific antagomirs may be required for antiangiogenic therapy. We found that the action of *miR-130a* is mediated through the modulation of *Jarid2* expression both in mouse and zebrafish, however, the possibility of other regulatory mechanisms can not be ruled out and warrant further study in the future. *Jarid2* is a member of the Jumonji C (JmjC) and Arid-domain protein family and serves as a transcriptional repressor by facilitating the binding of Polycomb Repressive Complex (PRC) 2 to the target genes [45, 46]. Based on our results, we proposed that *miR-130a* promotes the angiogenic response by inhibiting the suppressor such as *Jarid2* in the endothelium. Suppresion of *Jarid2* could switch on the activation of genes responsible for the angiogenic response; however, we realize that involvement of additional pathways cannot be ruled out (Fig 5P). It has been shown that the levels of *Jarid2* manifest the proper differentiation of ES cells and embryonic development as the *Jarid2-mutant* mice are embryonic lethal due to cardiovascular defects [46, 47]. *Tie2-Cre*-mediated deletion of *Jarid2* (*Jarid2*^*en*^) phenocopies the *Jarid2* knockout, indicating its essential role in the endothelial lineage during embryogenesis [46]. This study supports that fine-tuning the levels of *Jarid2* through *miR-130a* in the endothelial lineages is one of the mechanisms to control the angiogenic response during development.

## Supporting information

S1 Fig *Etv2-Cre*-mediated *Dicer* deletion results in vascular abnormalities. **A, B,** Representative images of whole-mount embryos (A) and quantification (B) of *Dicer*^*L/L*^ and *Etv2*^*Cre/+*^*;Dicer*^*L/L*^ embryos. Note the reduced embryos size in the *Etv2*^*Cre/+*^*;Dicer*^*L/L*^ embryos. **C, D,** Immunostaining using anti-CD31 antibodies of the transverse sections of *Dicer*^*L/L*^ and *Etv2*^*Cre/+*^*;Dicer*^*L/L*^ embryos at E9.5. Panel C’ and D’ shows higher magnification of the boxed area in panel C and D. **E-H,** Immunostaining using EYFP (green; E, G) and CD31 (red; F, H) antibodies of the parasagittal sections of *Dicer*^*L/L*^ and *Etv2*^*Cre/+*^*;Dicer*^*L/L*^ embryos at E9.5. Note the reduced vascular plexus in *Etv2*^*Cre/+*^*;Dicer*^*L/L*^. Nuclei were stained with DAPI (blue). Error bars indicate SEM (*p<0.05).
(TIF)

S2 Fig Induction of *miR-130a* has no impact on cellular death. Representative images of Annexin V-FITC labeled differentiating cells in the absence (-Dox) and presence (+Dox) of doxycline.
(TIF)

S3 Fig *miR-130a* regulates endothelial patterning *in vivo*. **A, B,** Brightfield microscopic images revealed no major changes in gross morphology of mismatch control-2 and *miR-130a* morphants. **C-F,** Lateral fluorescence images of Tg(*fli1a*:*EGFP*) zebrafish lines revealed defective vasculature in *miR-130a* morphants (white arrowheads) at 48 hpf (C, D) and 72 hpf (E, F) time periods. **G,** Quantitative analysis of the number of defective zebrafish embryos with perturbed inter-somitic vessels (ISVs) at 48 hpf and 72 hpf. Error bars indicate SEM (**p<0.01).
(TIF)

S4 Fig *miR-130a* regulates *Jarid2* expression. **A-F,** qPCR analysis of *HoxA5*, *Robo1*, *Robo2*, *Slit1*, *Timp2* and *Notch1* expression using RNA isolated from wild-type EBs, *miR-130a* iEBs and *miR-130a-null* EBs at d6 of differentiation. Error bars indicate SEM (*p<0.05).
(TIF)

S5 Fig *Jarid2* is expressed in the endothelial population. **A-D,** qPCR analysis of *Etv2*, *Tie2*, *CD31* and *Jarid2* expression using RNA isolated from the EYFP^-^ and EYFP^+^ cell populations using *Etv2-EYFP* transgenic mouse embryos at E8.5. Note that *Jarid2* is expressed in both EYFP^-^ and EYFP^+^ cell populations. **E,** Heatmap showing expression of *Pdgfra* and *Jarid2* obtained from microarray analysis of EYFP^-^ and EYFP^+^ cell populations from wild-type and Etv2^-/-^ embryos at E8.5. Note that the enrichment of *Jarid2* in the Etv2^-/-^ embryos is restricted to EYFP^+^ populations. Error bars indicate SEM (**p<0.01; *p<0.05).
(TIF)

S6 Fig *Jarid2* over-expression inhibits vascular development. **A,** Tube formation assay using HUVEC cells following transfection with the vector and *Jarid2* constructs, respectively. White arrow indicates the defective tube formation **B,** Quantitative analysis of the number of vascular tubes per field at 10x magnigfication. Note the decreased number of tubes following over-expression of *Jarid2*. **C,** Quantitative analysis of tube formation following co-injection of *Jarid2* and *miR-130a* mimics. Note the restoration of tube formation upon co-expression of *Jarid2* and *miR-130a* mimics. Error bars indicate SEM (*p<0.05).
(TIF)

S1 Table *miR-130a* target prediction. Top 100 common miR-130a predicted gene targets between mouse and zebrafish. List of the targets were ranked on the basis of the average context score.
(DOCX)
