# Supplementary figures and images for: Etv2-miR-130a-Jarid2 cascade regulates vascular patterning during embryogenesis
Source: PLoS One. 2017 Dec 12;12(12):e0189010. doi: 10.1371/journal.pone.0189010 (PMC5726724; doi:10.1371/journal.pone.0189010)

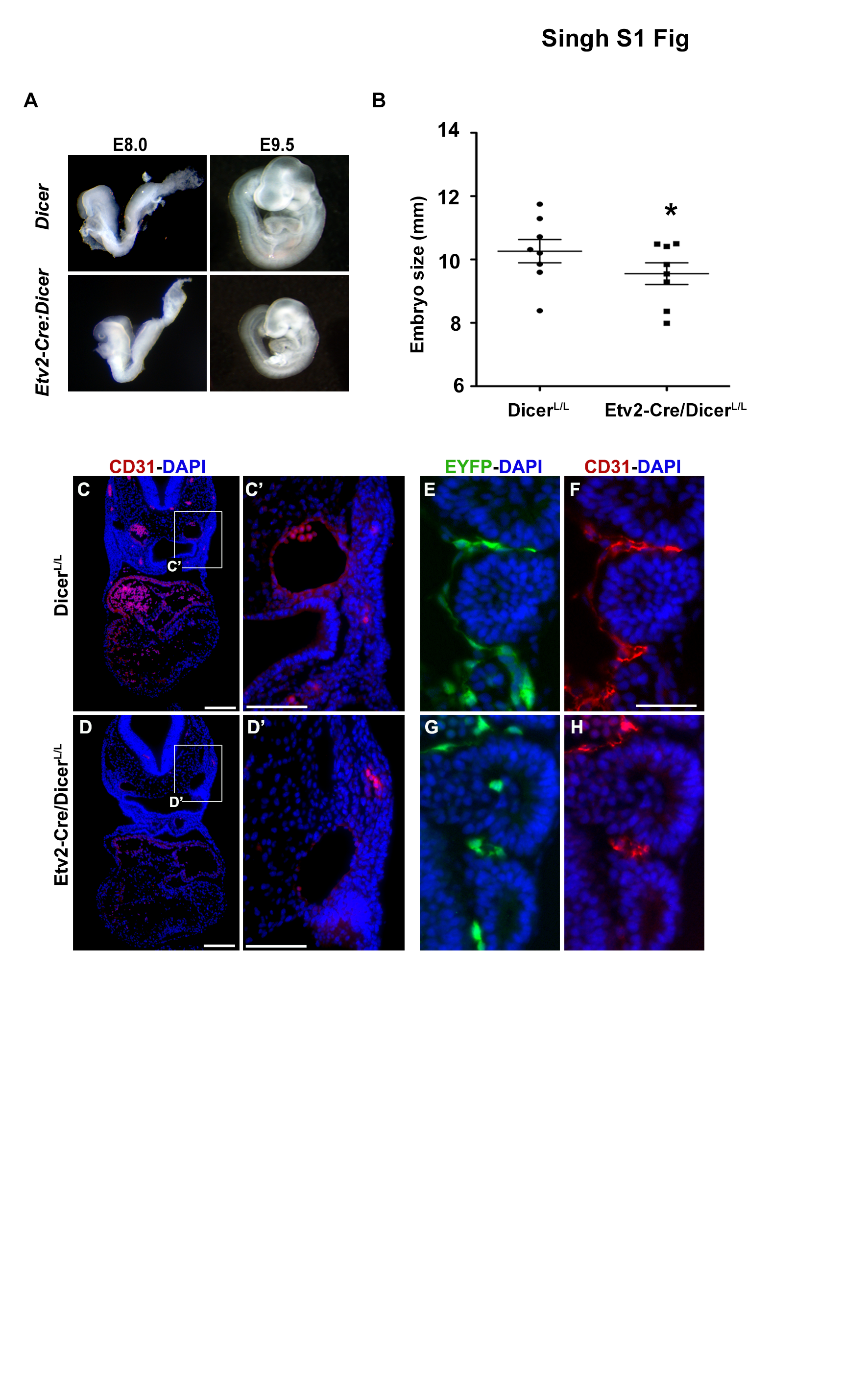

Supplement: S1 Fig — A, B, Representative images of whole-mount embryos (A) and quantification (B) of DicerL/L and Etv2Cre/+;DicerL/L embryos. Note the reduced embryos size in the Etv2Cre/+;DicerL/L embryos. C, D, Immunostaining using anti-CD31 antibodies of the transverse sections of DicerL/L and Etv2Cre/+;DicerL/L embryos at E9.5. Panel C’ and D’ shows higher magnification of the boxed area in panel C and D. E-H, Immunostaining using EYFP (green; E, G) and CD31 (red; F, H) antibodies of the parasagittal sections of DicerL/L and Etv2Cre/+;DicerL/L embryos at E9.5. Note the reduced vascular plexus in Etv2Cre/+;DicerL/L. Nuclei were stained with DAPI (blue). Error bars indicate SEM (*p<0.05). (TIF) [file pone.0189010.s001.tif]

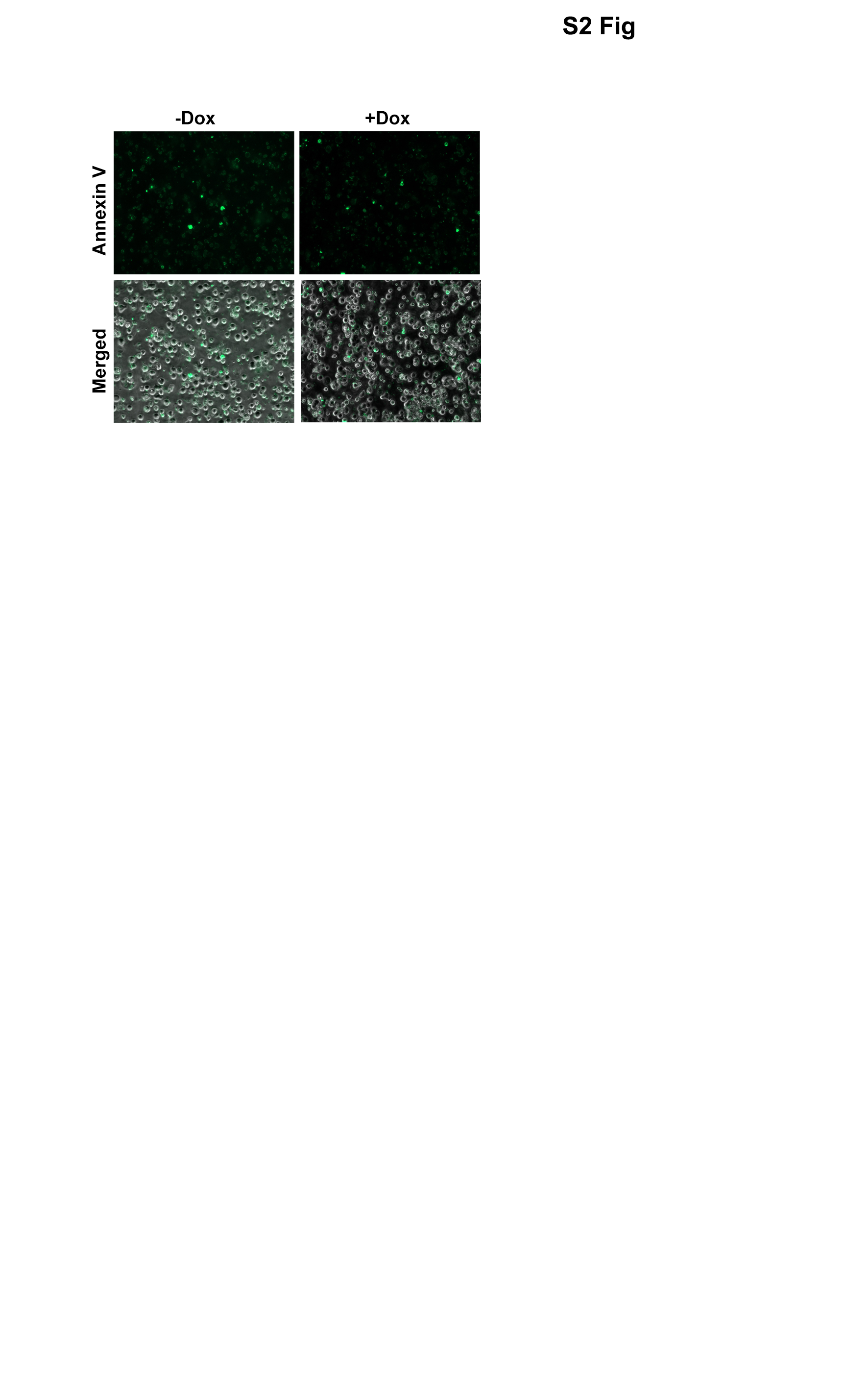

Supplement: S2 Fig — Representative images of Annexin V-FITC labeled differentiating cells in the absence (-Dox) and presence (+Dox) of doxycline. (TIF) [file pone.0189010.s002.tif]

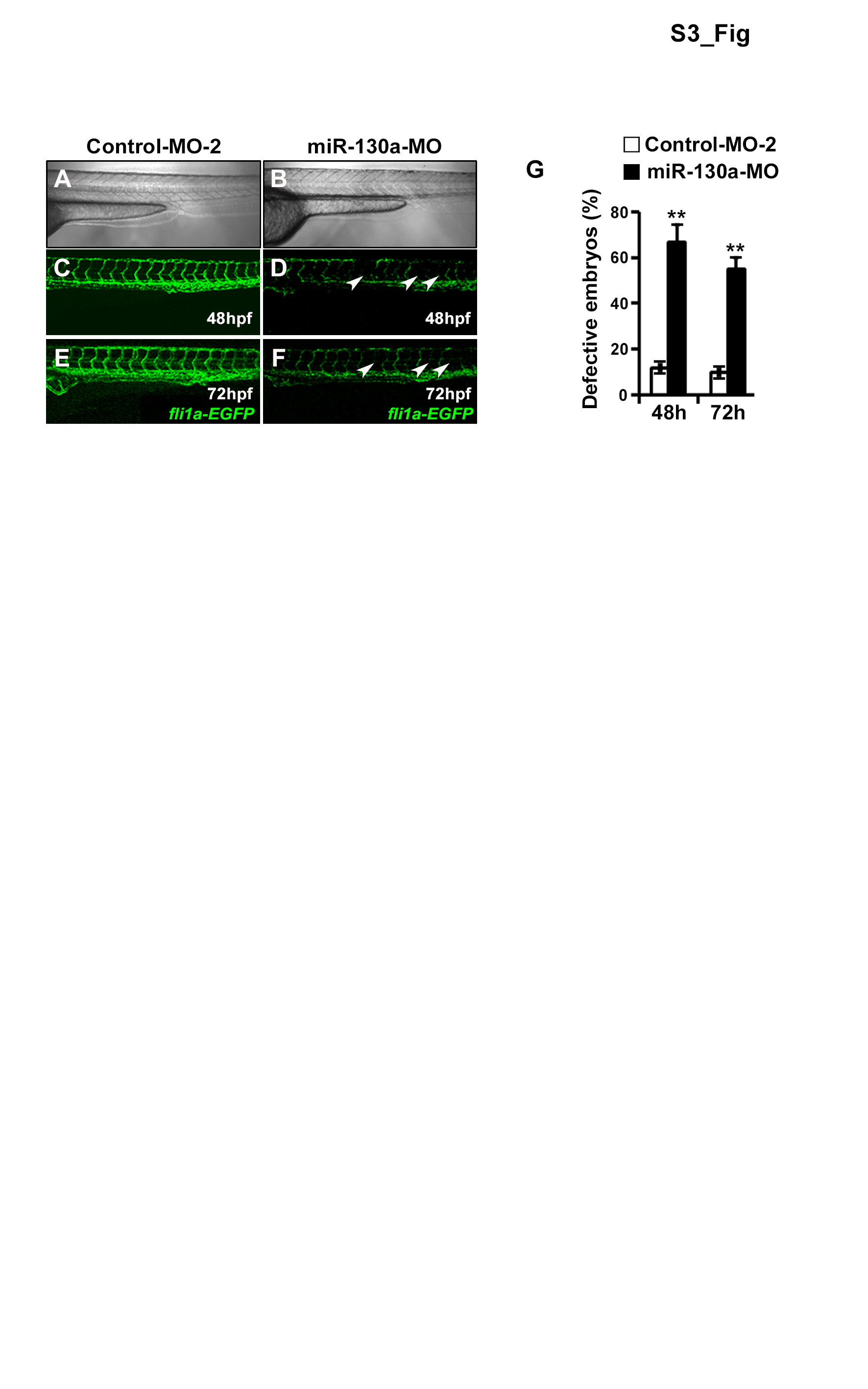

Supplement: S3 Fig — A, B, Brightfield microscopic images revealed no major changes in gross morphology of mismatch control-2 and miR-130a morphants. C-F, Lateral fluorescence images of Tg(fli1a:EGFP) zebrafish lines revealed defective vasculature in miR-130a morphants (white arrowheads) at 48 hpf (C, D) and 72 hpf (E, F) time periods. G, Quantitative analysis of the number of defective zebrafish embryos with perturbed inter-somitic vessels (ISVs) at 48 hpf and 72 hpf. Error bars indicate SEM (**p<0.01). (TIF) [file pone.0189010.s003.tif]

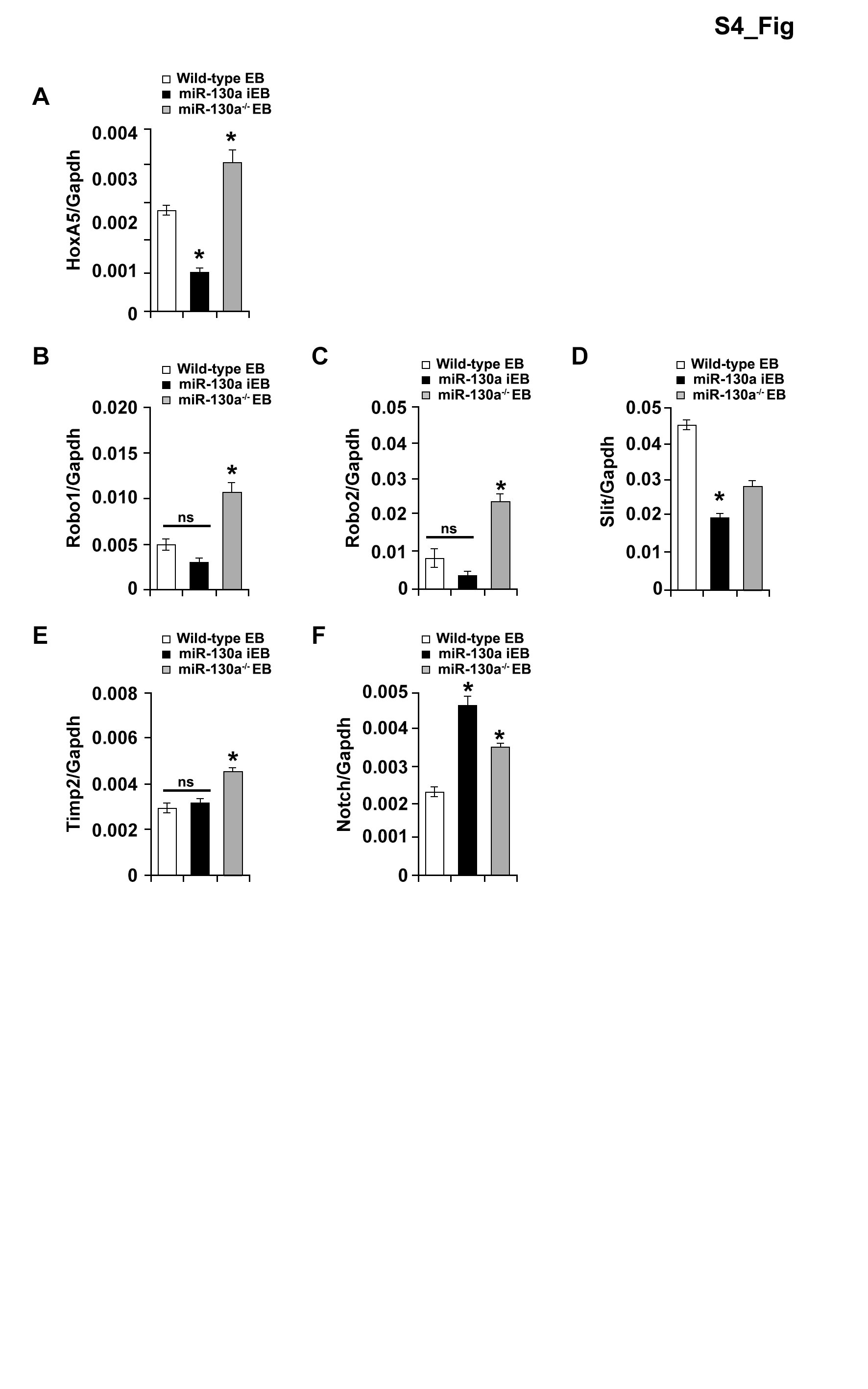

Supplement: S4 Fig — A-F, qPCR analysis of HoxA5, Robo1, Robo2, Slit1, Timp2 and Notch1 expression using RNA isolated from wild-type EBs, miR-130a iEBs and miR-130a-null EBs at d6 of differentiation. Error bars indicate SEM (*p<0.05). (TIF) [file pone.0189010.s004.tif]

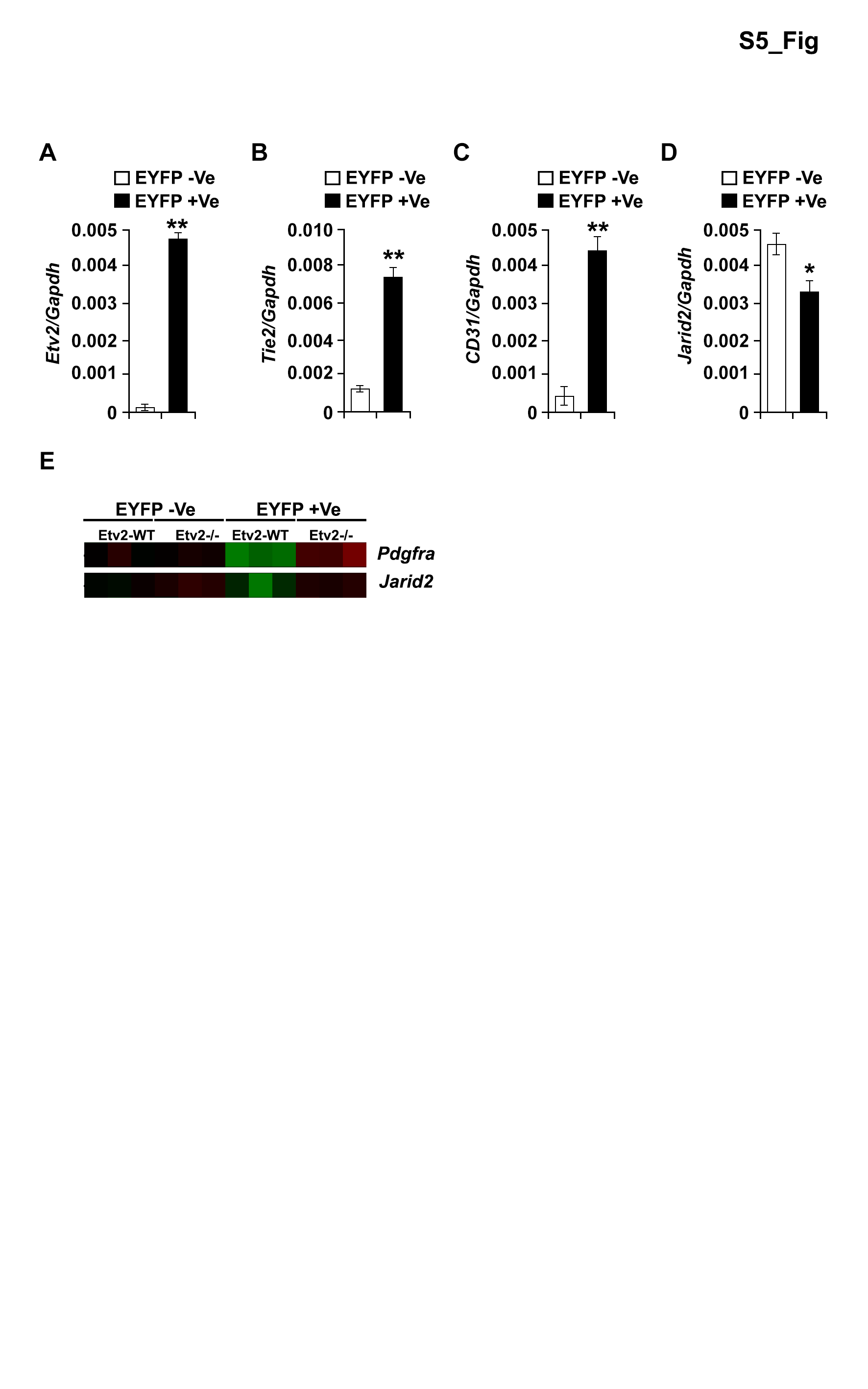

Supplement: S5 Fig — A-D, qPCR analysis of Etv2, Tie2, CD31 and Jarid2 expression using RNA isolated from the EYFP- and EYFP+ cell populations using Etv2-EYFP transgenic mouse embryos at E8.5. Note that Jarid2 is expressed in both EYFP- and EYFP+ cell populations. E, Heatmap showing expression of Pdgfra and Jarid2 obtained from microarray analysis of EYFP- and EYFP+ cell populations from wild-type and Etv2-/- embryos at E8.5. Note that the enrichment of Jarid2 in the Etv2-/- embryos is restricted to EYFP+ populations. Error bars indicate SEM (**p<0.01; *p<0.05). (TIF) [file pone.0189010.s005.tif]

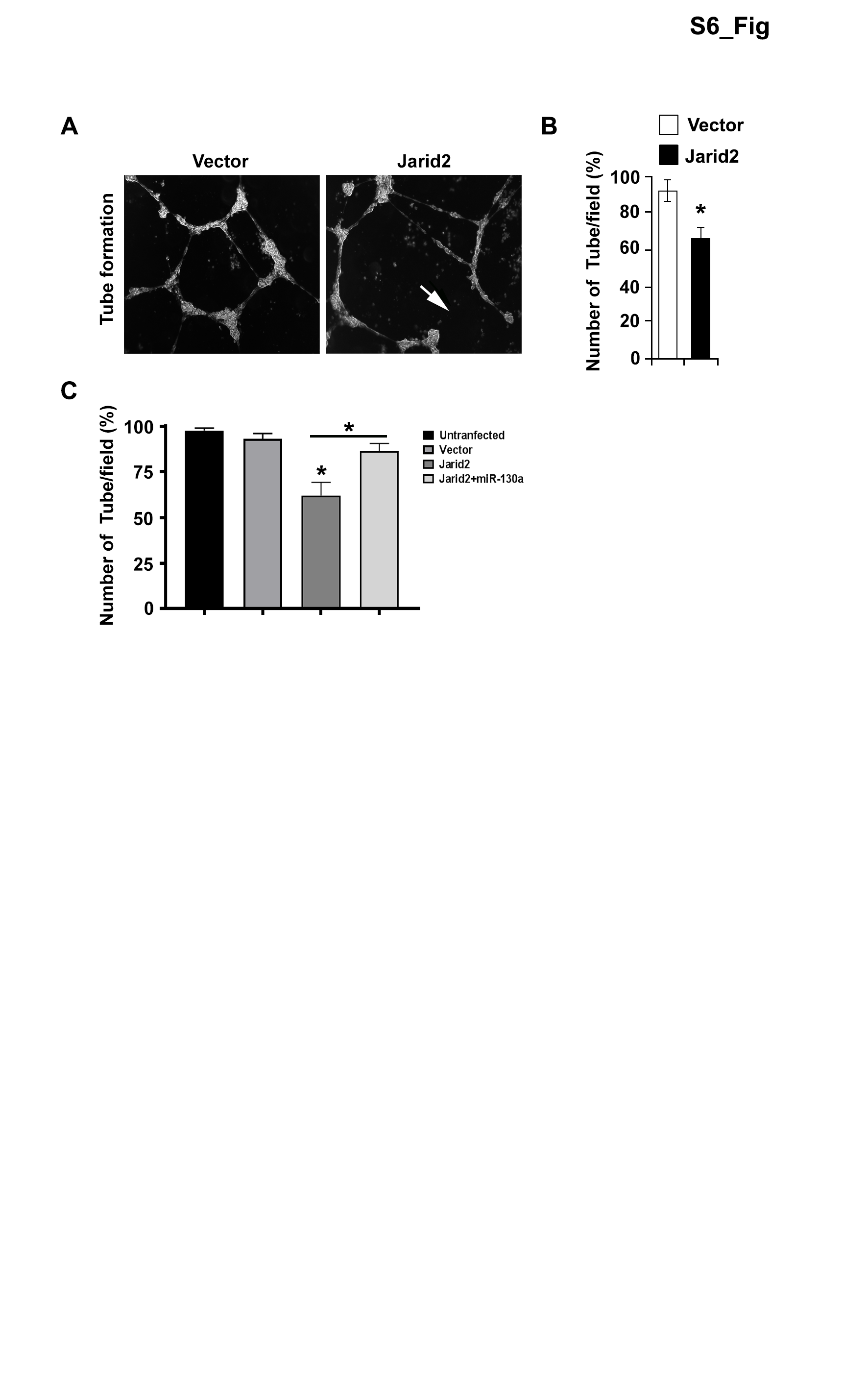

Supplement: S6 Fig — A, Tube formation assay using HUVEC cells following transfection with the vector and Jarid2 constructs, respectively. White arrow indicates the defective tube formation B, Quantitative analysis of the number of vascular tubes per field at 10x magnigfication. Note the decreased number of tubes following over-expression of Jarid2. C, Quantitative analysis of tube formation following co-injection of Jarid2 and miR-130a mimics. Note the restoration of tube formation upon co-expression of Jarid2 and miR-130a mimics. Error bars indicate SEM (*p<0.05). (TIF) [file pone.0189010.s006.tif]
