# Supplementary material for: Etv2-miR-130a-Jarid2 cascade regulates vascular patterning during embryogenesis
Source: PLoS One. 2017 Dec 12;12(12):e0189010. doi: 10.1371/journal.pone.0189010 (PMC5726724; doi:10.1371/journal.pone.0189010)
Supplement: S1 Table — Top 100 common miR-130a predicted gene targets between mouse and zebrafish. List of the targets were ranked on the basis of the average context score. (DOCX) [file pone.0189010.s007.docx]

**S1 Table:** Top 100 common miR-130a predicted gene targets between mouse and zebrafish. List of the targets were ranked on the basis of the average context score.

| mouse | | | zebrafish | | |  |
| --- | --- | --- | --- | --- | --- | --- |
| symbol | **accession** | **score** | **symbol** | **accession** | **score** | **rank** |
| Jarid2 | NM_001205043 | -0.47 | **jarid2a** | ENSDARG00000060925 | -0.73 | -0.62 |
| Acsl4 | NM_001033600 | -0.42 | **acsl4a** | ENSDARG00000004078 | -0.53 | -0.42 |
| Cpeb1 | NM_007755 | -0.41 | **cpeb1b** | ENSDARG00000008454 | -0.73 | -0.41 |
| Cbfb | NM_001161456 | -0.35 | **cbfb** | ENSDARG00000040917 | -0.36 | -0.35 |
| Rfx7 | NM_001033536 | -0.35 | **LOC560368** | ENSDARG00000077237 | -0.43 | -0.35 |
| Unc13a | NM_001029873 | -0.39 | **si:dkey-110c1.7** | ENSDARG00000061829 | -0.35 | -0.35 |
| Clcn5 | NM_016691 | -0.35 | **si:dkey-22f5.8** | ENSDARG00000019693 | -0.37 | -0.35 |
| Mybl1 | NM_008651 | -0.67 | **mybl1** | ENSDARG00000030999 | -0.34 | -0.34 |
| Calm2 | NM_007589 | -0.34 | **calm2b** | ENSDARG00000015050 | -0.34 | -0.34 |
| Gja1 | NM_010288 | -0.33 | **cx43** | ENSDARG00000041799 | -0.34 | -0.33 |
| Ptprg | NM_008981 | -0.38 | **ptprga** | ENSDARG00000045006 | -0.33 | -0.33 |
| Dennd1a | NM_146122 | -0.33 | **dennd1a** | ENSDARG00000014592 | -0.54 | -0.33 |
| Dll1 | NM_007865 | -0.3 | **dld** | ENSDARG00000020219 | -0.48 | -0.3 |
| Usp32 | NM_001029934 | -0.3 | **usp32** | ENSDARG00000061895 | -0.36 | -0.3 |
| Kmt2c | NM_001081383 | -0.29 | **kmt2cb** | ENSDARG00000075560 | -0.29 | -0.29 |
| Kmt2c | NM_001081383 | -0.29 | **kmt2ca** | ENSDARG00000079312 | -0.31 | -0.29 |
| Habp4 | NM_019986 | -0.29 | **zgc:103482** | ENSDARG00000025174 | -0.27 | -0.27 |
| Asxl2 | NM_172421 | -0.27 | **LOC100150267** | ENSDARG00000076501 | -0.36 | -0.27 |
| Rtn1 | NM_001007596 | -0.27 | **rtn1a** | ENSDARG00000006497 | -0.3 | -0.27 |
| Adcy7 | NM_001037723 | -0.3 | **adcy7** | ENSDARG00000060070 | -0.26 | -0.26 |
| Foxf2 | NM_010225 | -0.27 | **foxf2a** | ENSDARG00000017195 | -0.26 | -0.26 |
| Hoxb1 | NM_008266 | -0.26 | **hoxb1a** | ENSDARG00000008174 | -0.26 | -0.26 |
| Hbp1 | NM_153198 | -0.25 | **hbp1** | ENSDARG00000028517 | -0.26 | -0.25 |
| Btbd3 | NM_001025431 | -0.26 | **btbd3b** | ENSDARG00000087517 | -0.25 | -0.25 |
| 2310035C23Rik | NM_029349 | -0.25 | **zgc:66014** | ENSDARG00000038043 | -0.26 | -0.25 |
| Brwd1 | NM_001103179 | -0.27 | **brwd1** | ENSDARG00000074747 | -0.25 | -0.25 |
| Hecw2 | NM_001001883 | -0.25 | **hecw2** | ENSDARG00000063253 | -0.31 | -0.25 |
| Tmem170b | NM_001163572 | -0.26 | **LOC100536306** | ENSDARG00000087025 | -0.25 | -0.25 |
| Fam43a | NM_177632 | -0.27 | **fam43a** | ENSDARG00000043009 | -0.24 | -0.24 |
| Trim2 | NM_030706 | -0.29 | **trim2a** | ENSDARG00000031817 | -0.24 | -0.24 |
| Gpr137c | NM_027518 | -0.24 | **gpr137ba** | ENSDARG00000045289 | -0.52 | -0.24 |
| Epc2 | NM_172663 | -0.35 | **epc2** | ENSDARG00000007485 | -0.24 | -0.24 |
| Thsd7a | NM_001164805 | -0.24 | **thsd7a** | ENSDARG00000061479 | -0.28 | -0.24 |
| Chic1 | NM_009767 | -0.24 | **chic1** | ENSDARG00000053166 | -0.28 | -0.24 |
| Calm2 | NM_007589 | -0.34 | **calm3a** | ENSDARG00000044537 | -0.24 | -0.24 |
| Prkd3 | NM_001171004 | -0.23 | **prkd3** | ENSDARG00000079967 | -0.32 | -0.23 |
| Psd3 | NM_027626 | -0.28 | **psd3l** | ENSDARG00000053210 | -0.23 | -0.23 |
| Ankib1 | NM_001003909 | -0.22 | **ankib1** | ENSDARG00000076829 | -0.24 | -0.22 |
| Robo2 | NM_175549 | -0.23 | **robo2** | ENSDARG00000014891 | -0.21 | -0.21 |
| Ikzf4 | NM_011772 | -0.21 | **si:dkey-166n8.9** | ENSDARG00000037068 | -0.36 | -0.21 |
| Spopl | NM_001165997 | -0.24 | **spopla** | ENSDARG00000010563 | -0.21 | -0.21 |
| Cltc | NM_001003908 | -0.2 | **cltcb** | ENSDARG00000090716 | -0.2 | -0.2 |
| Grb10 | NM_001177629 | -0.2 | **grb10b** | ENSDARG00000035308 | -0.25 | -0.2 |
| Neurog1 | NM_010896 | -0.2 | **neurog1** | ENSDARG00000056130 | -0.25 | -0.2 |
| Lrp12 | NM_172814 | -0.22 | **lrp12** | ENSDARG00000059674 | -0.2 | -0.2 |
| Pdgfra | NM_001083316 | -0.2 | **pdgfra** | ENSDARG00000090703 | -0.26 | -0.2 |
| Cnot7 | NM_011135 | -0.2 | **cnot7** | ENSDARG00000032116 | -0.27 | -0.2 |
| Nbea | NM_030595 | -0.24 | **LOC565885** | ENSDARG00000010158 | -0.2 | -0.2 |
| Emx2 | NM_010132 | -0.24 | **emx2** | ENSDARG00000039701 | -0.19 | -0.19 |
| St8sia3 | NM_009182 | -0.19 | **st8sia3** | ENSDARG00000045301 | -0.39 | -0.19 |
| Pex5l | NM_001163516 | -0.26 | **pex5la** | ENSDARG00000022518 | -0.19 | -0.19 |
| D230025D16Rik | NM_145604 | -0.19 | **si:dkey-102f14.5** | ENSDARG00000041560 | -0.3 | -0.19 |
| Kbtbd8 | NM_001008785 | -0.3 | **kbtbd8** | ENSDARG00000070698 | -0.19 | -0.19 |
| Abca1 | NM_013454 | -0.39 | **abca1b** | ENSDARG00000079009 | -0.19 | -0.19 |
| Ddhd2 | NM_028102 | -0.28 | **ddhd2** | ENSDARG00000076765 | -0.19 | -0.19 |
| Tmem63b | NM_198167 | -0.19 | **tmem63ba** | ENSDARG00000003836 | -0.2 | -0.19 |
| Dynll2 | NM_001168471 | -0.19 | **dynll2b** | ENSDARG00000005172 | -0.46 | -0.19 |
| AU040320 | NM_133886 | -0.19 | **si:ch211-193k19.1** | ENSDARG00000035660 | -0.18 | -0.18 |
| Pou4f1 | NM_011143 | -0.38 | **pou4f1** | ENSDARG00000005559 | -0.18 | -0.18 |
| Fam222a | NM_001004180 | -0.18 | **fam222a** | ENSDARG00000090797 | -0.19 | -0.18 |
| Tsc1 | NM_022887 | -0.3 | **tsc1a** | ENSDARG00000026048 | -0.17 | -0.17 |
| Gclc | NM_010295 | -0.17 | **gclc** | ENSDARG00000013095 | -0.19 | -0.17 |
| Itpr1 | NM_010585 | -0.21 | **itpr1** | ENSDARG00000074149 | -0.17 | -0.17 |
| Timp2 | NM_011594 | -0.17 | **timp2a** | ENSDARG00000061226 | -0.2 | -0.17 |
| Lgalsl | NM_173752 | -0.41 | **lgalsla** | ENSDARG00000059060 | -0.17 | -0.17 |
| Fstl5 | NM_178673 | -0.27 | **fstl5** | ENSDARG00000040198 | -0.17 | -0.17 |
| Plekhg5 | NM_001004156 | -0.17 | **LOC796404** | ENSDARG00000089038 | -0.26 | -0.17 |
| Lrp2 | NM_001081088 | -0.17 | **lrp2a** | ENSDARG00000060649 | -0.19 | -0.17 |
| Lnpep | NM_172827 | -0.17 | **lnpep** | ENSDARG00000014403 | -0.33 | -0.17 |
| Syt2 | NM_009307 | -0.23 | **LOC559637** | ENSDARG00000014169 | -0.17 | -0.17 |
| Slmap | NM_032008 | -0.42 | **slmapa** | ENSDARG00000054458 | -0.17 | -0.17 |
| E2f2 | NM_177733 | -0.2 | **LOC568846** | ENSDARG00000079233 | -0.17 | -0.17 |
| Hipk3 | NM_001145824 | -0.18 | **hipk3b** | ENSDARG00000062082 | -0.17 | -0.17 |
| Ston2 | NM_175367 | -0.31 | **ston2** | ENSDARG00000057452 | -0.17 | -0.17 |
| Inhbb | NM_008381 | -0.21 | **inhbb** | ENSDARG00000040777 | -0.16 | -0.16 |
| Tgfb2 | NM_009367 | -0.16 | **tgfb2** | ENSDARG00000027087 | -0.25 | -0.16 |
| Cltc | NM_001003908 | -0.2 | **cltca** | ENSDARG00000043493 | -0.16 | -0.16 |
| Mtmr12 | NM_172958 | -0.16 | **mtmr12** | ENSDARG00000059817 | -0.35 | -0.16 |
| Memo1 | NM_133771 | -0.21 | **memo1** | ENSDARG00000010823 | -0.15 | -0.15 |
| Trim3 | NM_018880 | -0.15 | **trim3a** | ENSDARG00000063711 | -0.17 | -0.15 |
| Sphk2 | NM_001172561 | -0.28 | **sphk2** | ENSDARG00000069893 | -0.15 | -0.15 |
| Rab5a | NM_025887 | -0.58 | **rab5aa** | ENSDARG00000018602 | -0.15 | -0.15 |
| Clock | NM_007715 | -0.14 | **clock** | ENSDARG00000011703 | -0.14 | -0.14 |
| Lrig1 | NM_008377 | -0.14 | **lrig1** | ENSDARG00000075625 | -0.21 | -0.14 |
| Tmcc1 | NM_177412 | -0.14 | **zgc:153972** | ENSDARG00000060954 | -0.15 | -0.14 |
| Katnbl1 | NM_024254 | -0.14 | **katnbl1** | ENSDARG00000042522 | -0.19 | -0.14 |
| Slitrk2 | NM_001161431 | -0.14 | **slitrk2** | ENSDARG00000006636 | -0.2 | -0.14 |
| Tmem9b | NM_020050 | -0.15 | **tmem9b** | ENSDARG00000025693 | -0.14 | -0.14 |
| Klf13 | NM_021366 | -0.17 | **klf13** | ENSDARG00000061368 | -0.14 | -0.14 |
| Skp1a | NM_011543 | -0.18 | **skp1** | ENSDARG00000003151 | -0.14 | -0.14 |
| Eogt | NM_175313 | -0.24 | **LOC798087** | ENSDARG00000022853 | -0.14 | -0.14 |
| Cds1 | NM_173370 | -0.31 | **si:ch211-168m18.1** | ENSDARG00000019549 | -0.14 | -0.14 |
| Dicer1 | NM_148948 | -0.13 | **dicer1** | ENSDARG00000001129 | -0.44 | -0.13 |
| Larp4 | NM_001024526 | -0.13 | **LOC797570** | ENSDARG00000078401 | -0.5 | -0.13 |
| Lrch1 | NM_001033439 | -0.13 | **si:ch211-260b17.8** | ENSDARG00000078778 | -0.34 | -0.13 |
| Hoxb3 | NM_001079869 | -0.12 | **hoxb3a** | ENSDARG00000029263 | -0.15 | -0.12 |
| Itpkb | NM_001081175 | -0.13 | **itpkb** | ENSDARG00000010059 | -0.12 | -0.12 |
| Nhlh2 | NM_178777 | -0.12 | **nhlh2** | ENSDARG00000025495 | -0.22 | -0.12 |
